# Effects of ocean acidification on Antarctic marine organisms: A meta‐analysis

**DOI:** 10.1002/ece3.6205

**Published:** 2020-04-16

**Authors:** Alyce M. Hancock, Catherine K. King, Jonathan S. Stark, Andrew McMinn, Andrew T. Davidson

**Affiliations:** ^1^ Institute for Marine and Antarctic Studies University of Tasmania Battery Point TAS Australia; ^2^ Antarctic Gateway Partnership Battery Point TAS Australia; ^3^ Antarctic Climate & Ecosystems Cooperative Research Centre Battery Point TAS Australia; ^4^ Australian Antarctic Division Kingston TAS Australia

**Keywords:** bacteria, climate change, CO_2_, fish, invertebrates, macroalgae, pH, phytoplankton, Southern Ocean

## Abstract

Southern Ocean waters are among the most vulnerable to ocean acidification. The projected increase in the CO_2_ level will cause changes in carbonate chemistry that are likely to be damaging to organisms inhabiting these waters. A meta‐analysis was undertaken to examine the vulnerability of Antarctic marine biota occupying waters south of 60°S to ocean acidification. This meta‐analysis showed that ocean acidification negatively affects autotrophic organisms, mainly phytoplankton, at CO_2_ levels above 1,000 μatm and invertebrates above 1,500 μatm, but positively affects bacterial abundance. The sensitivity of phytoplankton to ocean acidification was influenced by the experimental procedure used. Natural, mixed communities were more sensitive than single species in culture and showed a decline in chlorophyll *a* concentration, productivity, and photosynthetic health, as well as a shift in community composition at CO_2_ levels above 1,000 μatm. Invertebrates showed reduced fertilization rates and increased occurrence of larval abnormalities, as well as decreased calcification rates and increased shell dissolution with any increase in CO_2_ level above 1,500 μatm. Assessment of the vulnerability of fish and macroalgae to ocean acidification was limited by the number of studies available. Overall, this analysis indicates that many marine organisms in the Southern Ocean are likely to be susceptible to ocean acidification and thereby likely to change their contribution to ecosystem services in the future. Further studies are required to address the poor spatial coverage, lack of community or ecosystem‐level studies, and the largely unknown potential for organisms to acclimate and/or adapt to the changing conditions.

## Graphical abstract

Antarctic waters are among the most vulnerable in the world to ocean acidification due to their cold temperatures, naturally low levels of calcium carbonate and upwelling that brings deep CO_2_‐rich waters to the surface. This meta‐analysis demonstrates groups of Antarctic marine biota in waters south of 60°S have a range of tolerances to ocean acidification. Invertebrates and phytoplankton showed negative effects above 500 μatm and 1,000 μatm CO_2_, respectively, while bacteria appear tolerant to elevated CO_2_.

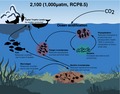

## 1 INTRODUCTION

Much of the anthropogenic atmospheric carbon dioxide (CO_2_) emissions is estimated to have been absorbed by the world's oceans leading to significant changes in seawater carbonate chemistry and reduced pH (Doney et al., 2012; Frölicher et al., 2015; Gattuso & Hansson, 2011; Gattuso & Lavigne, 2009; Le Quéré et al., 2018; Raven & Falkowski, 1999; Sabine et al., 2004). Polar waters are particularly vulnerable to ocean acidification due to the increased solubility of CO_2_ at lower temperatures, naturally low levels of calcium carbonate, and a lower buffering capacity (Byrne, Mecking, Feely, & Liu, 2010; Dore, Lukas, Sadler, Church, & Karl, 2009; Ishii et al., 2011; Lovenduski, Gruber, Doney, & Lima, 2007; Orr, Epitalon, & Gattuso, 2014; Orr et al., 2005). High levels of upwelling also occur in polar waters, bringing deep CO_2_‐rich waters to the surface (Doney, Fabry, Feely, & Kleypas, 2009; Fabry, McClintock, Mathis, & Grebmeier, 2009; Hauri, Friedrich, & Timmermann, 2016; Le Quéré et al., 2007; McNeil & Matear, 2008; Negrete‐García, Lovenduski, Hauri, Krumhardt, & Lauvset, 2019; Orr et al., 2005). In addition, coastal waters have increased levels of terrestrial freshwater input during summer, and lack of photosynthetic driven draw‐down of CO_2_ during the light‐limited winter months (Gibson & Trull, 1999; Gruber et al., 2012; Kapsenberg, Kelley, Shaw, Martz, & Hofmann, 2015; McNeil, Tagliabue, & Sweeney, 2010; Melzner et al., 2013; Roden, Shadwick, Tilbrook, & Trull, 2013). Thus, organisms living in the Southern Ocean and nearshore Antarctic waters are likely to be exposed to potentially damaging pH and calcium carbonate conditions earlier than elsewhere in the world's oceans.

The pH of Southern Ocean surface waters has already decreased by ≈0.1 pH units and is predicted to decrease by a further ≈0.3 units by 2100 under the IPCC RCP8.5 “business as usual” scenario, equating to surface water CO_2_ levels >1,000 μatm (Kawaguchi et al., 2013; McNeil & Matear, 2007, 2008). Coupled intercomparison projects (CMIP5) show that low latitude waters could experience a decrease in pH from pre‐industrial levels of 8.17–7.77 by 2100, or 880 to 930 μatm CO_2_ (Hartin, Bond‐Lamberty, Patel, & Mundra, 2016). Only under the lowest CO_2_ emissions scenario with stringent mitigation will these changes be avoided (IPCC, 2019). In addition, there is a large natural seasonal variability in pH. During winter, pH is reduced and the concentration of carbonate ions is naturally low in the Southern Ocean and nearshore Antarctic waters. In contrast, during summer, pH and carbonate ion concentrations are higher due to changes in seasonal mixing and biological productivity (Gibson & Trull, 1999; Kapsenberg et al., 2015; McNeil & Matear, 2007; McNeil et al., 2010; Roden et al., 2013). Future predictions of acidification indicate that winter surface waters south of 60°S could reach 1,000 μatm by 2100 when seasonal CO_2_ cycles are incorporated into model predictions, and areas of the Southern Ocean could experience undersaturation in aragonite by as early as 2030 under the IPCC RCP8.5 (Kawaguchi et al., 2013; McNeil & Matear, 2008; McNeil & Sasse, 2016). Aragonite undersaturation could occur in ≈30% of the Southern Ocean by 2060 and >70% by 2100 (Hauri et al., 2016). The duration of this surface undersaturation is predicted to increase from 1 to 6 months within the next 20 years (Hauri et al., 2016). Surface waters of the Southern Ocean are even predicted to become undersaturated in calcite (the more stable form of calcium carbonate) by 2095, which is several decades earlier than elsewhere in the world's oceans (Hauri et al., 2016; Negrete‐García et al., 2019). The implications of the decrease in the pH and early undersaturation could be significant for many organisms within the Southern Ocean (Wittmann & Pörtner, 2013). Thus, these waters have been suggested to serve as a bellweather for the impacts of ocean acidification globally (Fabry et al., 2009; Negrete‐García et al., 2019).

Meta‐analyses are a useful tool to synthesize the results of multiple studies, to provide meaningful comparisons to determine the overall state of knowledge in an area and highlight areas of uncertainty. A number of meta‐analyses have focused on the global effects of ocean acidification on marine organisms (i.e., Kroeker, Kordas, Crim, & Singh, 2010; Kroeker et al., 2013; Wittmann & Pörtner, 2013). These analyses have found that ocean acidification has a negative effect on the survival, calcification, growth, and reproduction of marine biota but with significant variation in sensitivity among taxonomic groups. All calcifying organisms except crustaceans had larger negative responses than noncalcifying organisms (Kroeker et al., 2010, 2013). Larval and early life stages are also highly sensitive, particularly in mollusks (Kroeker et al., 2013). No meta‐analysis has yet been conducted to specifically examine the effects of ocean acidification on marine organisms in the Southern Ocean and nearshore Antarctic waters, despite the high vulnerability of these regions.

This study presents a comprehensive meta‐analysis on the effect of altered seawater carbonate chemistry on marine organisms south of 60°S. It aims to provide a synthesis of current understanding in the area and highlights knowledge gaps and future research directions. As part of this meta‐analysis, this study investigates whether there are tipping points in the response of marine organisms to ocean acidification and therefore predict at what future CO_2_ concentration and time organisms inhabiting the Southern Ocean and nearshore Antarctic ecosystems may be significantly affected by ocean acidification.

## 2 METHODS

### 2.1 Data selection and suitability criteria

Comprehensive database searches were conducted to compile all English language, peer‐reviewed journal articles and literature reviews that investigated the effect of altered seawater carbonate chemistry on Southern Ocean and/or Antarctic marine organisms. Searches were conducted using the ISI Web of Science [v.5.27.2], Scopus, and ASFA (Aquatic Sciences and Fisheries Abstracts) databases using the search terms given in Table S1 (all searches were completed by the June 2019). The OA‐ICC bibliographic database (https://www.iaea.org/ocean-acidification/) was also searched along with literature cited in bibliographies of all the articles and reviews identified in the literature search. Articles were then screened to only include studies that manipulated the carbonate chemistry to investigate the effects of ocean acidification on marine organisms collected, or experiments conducted south of 60°S. Some studies included a number of species and/or locations, some of which were in the Arctic or north of 60°S. For these, only data from south of 60°S were included in the meta‐analysis and each species was included separately. After this manual screening, 64 papers remained for inclusion in the meta‐analysis. For studies that included more than one experiment or species, each individual experiment/species was included in the analysis. A number of studies investigated the effects of multiple stressors (i.e., ocean acidification and warming). The data associated with the effect of ocean acidification for these studies were included only if all other stressors were at ambient levels. For example, Torstensson, Hedblom, Björk, Chierici, and Wulff (2015) investigated the effect of elevated CO_2_ and temperature on an Antarctic diatom, and for this study, only the ambient temperature treatments were used. Any studies that did not have a treatment approximate to ambient levels for the costressor were not included in the meta‐analysis, such as those that investigated the effects of elevated CO_2_ and temperature but included no ambient temperature treatment. Only studies conducted over weeks (up to month) were included in the analysis, and longer experiments studying chronic acclimation and/or adaptation responses of organisms to ocean acidification were excluded (only three papers, Trimborn, Thoms, Petrou, Kranz, & Rost, 2014 and Torstensson et al., 2015 both investigating long‐term effects of elevated CO_2_ on phytoplankton cultures, and Ericson et al., 2018 investigating the long‐term effect of ocean acidification on Antarctic krill). Screening for experimental procedure used within the studies was not performed due to the limited number of studies available to include in the meta‐analysis.

### 2.2 CO_2_ treatment levels and organism responses

The main aim of this study was to determine tolerance of Antarctic marine organisms to increased CO_2_ and if there were tipping points in their response, therefore analyses were conducted over a range of CO_2_ treatments above ambient. Ambient was identified as the CO_2_ treatment reported in the study as being either the “control” or “ambient” (all ambient levels were below ≈500 μatm). The CO_2_ treatment levels used in studies were separated into the following CO_2_ categories for the meta‐analysis: pre‐industrial (found in phytoplankton studies only), 500–800, 801–1,000, 1,001–1,500, 1,501–2,000, and >2,000 μatm. If studies reported the level of CO_2_ in units other than μatm, or if pH was reported, then the CO_2_ levels were converted into μatm of CO_2_ using CO_2_Sys v2.1.xls (Pierrot, Lewis, & Wallace, 2006). The data required for the conversion were reported within all papers except for two, and these were removed from the meta‐analysis as the corresponding author for these papers could not be contacted. If a study did not specify an ambient/control level, then the CO_2_ treatment closest to the ambient level at time of sample collection was used. Any studies using an ambient CO_2_ treatment which substantially exceeded 500 μatm were excluded from the meta‐analysis. If a study included multiple treatments in one CO_2_ category, then the highest treatment was used in the meta‐analysis, except for the pre‐industrial CO_2_ category in which the lowest was used. Study mean, standard error, and sample size for each experiment/species, biological response (described below), and CO_2_ category were used in the meta‐analysis. Data reported in graphs were extracted using the software WebPlotDigitizer v1.7.9 (Rohatgi). A number of studies reported biological response data in boxplots with medians and interquartile ranges rather than reporting the mean, standard error, and sample size. These were converted using the method of Luo, Wan, Liu, and Tong (2018a) for the estimation of mean and Luo, Wan, Liu, and Tong (2018b) for estimating the standard error. The equations were as follows:

$$\text{Estimation of mean}:\;\tilde{X}\left(w\right)\approx \left(0.7+\frac{0.39}{n}\right)\frac{q_{1}+q_{2}}{2}+\left(0.3-\frac{0.39}{n}\right)m.$$

$$\text{Estimation of standard error:}\;\;S\approx \frac{q_{3}-q_{1}}{\eta }\;\text{where}\;\eta =\eta \left(n\right)=2\Phi^{-1}\left[\frac{0.75n-0.125}{n+0.25}\right].$$

The final number of studies included in the meta‐analysis was 60 after manual screening and removal of studies that did not meet the above criteria (full list in Document S1).

Data for several taxonomic groups were included in the analysis, including prokaryotes; bacteria, autotrophic eukaryotes; phytoplankton and macroalgae, and heterotrophic eukaryotes; invertebrates and fish (Figures S1–S5). The invertebrates included amphipods (Arthropoda; Crustacea; Amphiphoda), krill (Arthropoda; Crustaceae; Euphausiacea), brachiopods (Brachiopoda), sea stars (Echinodermata; Asteroidea), sea urchins (Echinodermata; Echinoidea), bivalves (Mollusca; Bivalvia), pteropods (Mollusca; Gastropoda; Orthogastropoda; Opisthobranchia; Pteropoda), limpets (Mollusca; Gastropoda; Patellogastropoda), sea snails (Mollusca; Gastropoda; Orthogastropoda; Mesogastropoda; Trochidae), and nemertean worms (Nemertea). The effects of ocean acidification on different biological responses from each taxonomic group were investigated (Table 1). While most studies were conducted on a single species, a number of phytoplankton studies used natural or mixed‐species communities. These multispecies studies were included in the overall meta‐analysis. Further analyses were then conducted separating single species and multispecies phytoplankton studies to compare the difference in responses.

**TABLE 1:** Biological responses used for each taxonomic group in the meta‐analysis

Taxonomic group	Biological response
Prokaryotes	
Bacteria	Abundance
	Productivity
Autotrophic eukaryotes	
Phytoplankton	Chlorophyll *a* concentration (Chl *a*)
	Net primary productivity (NPP)
	Gross primary productivity (GPP)
	Effective quantum yield (*F*_v_/*F*_m_)
	Relative electron transport rate (rETR)
	Extracellular carbonic anhydrase activity (eCA)
	Total fatty acid content (fatty acids)
	Particulate organic carbon concentration (POC)
	Growth rate^a^
Macroalgae	Chlorophyll *a* concentration (Chl *a*)
	Effective quantum yield (*F*_v_/*F*_m_)
	Relative electron transport rate (rETR)
	Protein content (protein)
	Growth rate
Heterotrophic eukaryotes	
Invertebrates	Development (fertilization rate or normal development
	Shell state (adult calcification or dissolution rate)
	Growth rate (in adults)
	Survival (in adults)
	Behavior (adult maximal escape speeds)
	Feeding (adult consumption rate or energy absorbed)
Fish	Survival (of embryos)
	Oxygen (O_2_) consumption rates (in adults)
	Cellular aerobic potential, measured by citrate synthase enzyme activity in adults (CS)
	Growth rate (in adults)
	Heart rate (in adults)

^a^ If cell concentration was reported, then growth rate was calculated using $\mu =\frac{1}{t}\ln\left(\frac{N_{t}}{N_{0}}\right)$ (Pearce et al., 2007).

### 2.3 Data analysis

The data were analyzed following the methods of Kroeker et al. (2010) and Kroeker et al. (2013). For each biological response in each CO_2_ category, the ln‐transformed response ratio to ocean acidification was calculated for each study (or experiment/species within a study) using the formula:

$$\text{LnRR}=\ln\left(R\right)=\ln\left({\bar{X}}_{E}\right)-\ln\left({\bar{X}}_{C}\right)$$

where ${\bar{X}}_{E}$ and ${\bar{X}}_{C}$ are the mean value for the biological response in the CO_2_ experimental treatment and the ambient (control) treatment, respectively (Hedges, Gurevitch, & Curtis, 1999). The log response ratio (LnRR) was used due to its robustness to small sample sizes and its ability to detect true effects (Lajeunesse & Forbes, 2003). For all means, an increase in the mean is a positive response; that is, increased growth rate between control and experimental treatments is a positive response. If this was not true, that is, increase in dissolution of shells is a negative response, the mean was made negative. Thus, a positive response ratio represents a positive response of the organisms to ocean acidification, and a negative response ratio represents a negative effect of ocean acidification. The larger the response ratio (either positive or negative), the stronger the response and the effect of ocean acidification.

These response ratios (LnRR) were then weighted by the inverse of the effect size variance (*v*), calculated by their associated standard error and sample size using:

$$v=\frac{{\left(S_{E}\right)}^{2}}{n_{E}{\bar{X}}_{E}^{2}}+\frac{{\left(S_{C}\right)}^{2}}{n_{C}{\bar{X}}_{C}^{2}}$$

where $S_{E}$ and $S_{C}$ are the standard error for the experimental treatment mean and ambient (control) treatment mean, respectively, and *n*_E_ and *n*_C_ are the sample sizes for the experimental mean and ambient (control) mean, respectively (Hedges & Olkin, 1985). As above ${\bar{X}}_{E}$ and ${\bar{X}}_{C}$ are the mean value for the biological response in the CO_2_ experimental treatment and the ambient (control) treatment, respectively. This weighting ensured that studies with a lower standard error and higher sample size (data with greater certainty) were weighted more heavily in the analysis than those with higher standard error and lower sample size (lower certainty). The meta‐analysis was conducted on both weighted and unweighted response ratios, and there was no significant difference between the results; therefore, like Kroeker et al. (2010) and Kroeker et al. (2013), weighted analyses are presented.

Statistical analyses were conducted using the R v.1.0.136 (R Core Team, 2016) and the add‐on package “Metafor” (Viechtbauer, 2010). A random‐effects model was used to estimate a summary response ratio for each biological response in each CO_2_ category, weighted by the inverse‐variance weights (*v*). A random‐effects model was used as it accounts for both random sampling variation in each study and the variation among studies in estimating the effect size. In this model, the true variation in effect size is calculated by the between‐study variance (using the ln‐transformed response ratios, LnRR), with each study weighted by the inverse sum of the individual study variance (*v*). Restricted maximum‐likelihood estimator (REML) was used to estimate the amount of (residual) heterogeneity (*Q*), which tests whether the variability in the effect size is larger than would be expected based on sampling variance alone (Brown & Kempton, 1994). Statistical significance of the mean response ratio was based on the 95% confidence interval on the estimated summary response ratio (Viechtbauer, 2010).

Based on the availability of studies, organisms were assigned to functional groups at increasing levels of taxonomic resolution and the summary response ratios of each of these groups were determined for each CO_2_ category. The groups were:

1. Prokaryotes (bacteria), eukaryotic autotrophs (phytoplankton and macroalgae), and eukaryotic heterotrophs (invertebrates and fish), with all biological responses combined.
2. Bacteria, phytoplankton, macroalgae, invertebrates, and fish, with all biological responses combined.
3. Bacteria, phytoplankton, and invertebrates for each biological response. Due to the lack of studies, macroalgae and fish biological responses were investigated with all CO_2_ categories combined and for each macroalgal species with all biological responses and CO_2_ categories combined.
4. Single species‐ versus community‐level biological responses of phytoplankton.

## 3 RESULTS

**FIGURE 1:**
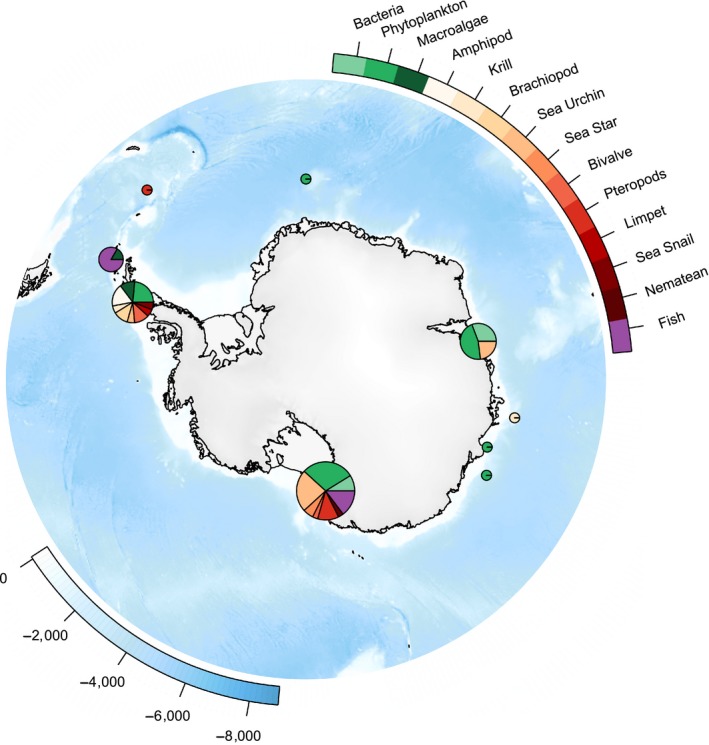
All studies on the effect of ocean acidification on marine organisms south of 60°S included in the meta‐analysis. Pie charts display the relative number of studies per organism at that location. The size of the pie reflects the total number of studies at that location. Figure created using the R package “SOmap” (Maschette, Sumner, & Raymond, 2018)

Sixty papers on the effect of altered carbonate chemistry on marine organisms south of 60°S were identified that met all the required selection criteria for inclusion in the meta‐analysis (list of papers included in Document S1). The majority of these studies were conducted in nearshore Antarctic waters, with only four offshore, open Southern Ocean waters studies (Figure 1). It is also apparent that most studies were conducted in areas surrounding the Antarctic Peninsula (particularly King George Island), the Ross Sea and Prydz Bay, East Antarctica with few studies in the oligotrophic waters of the Southern Ocean.

**FIGURE 2:**
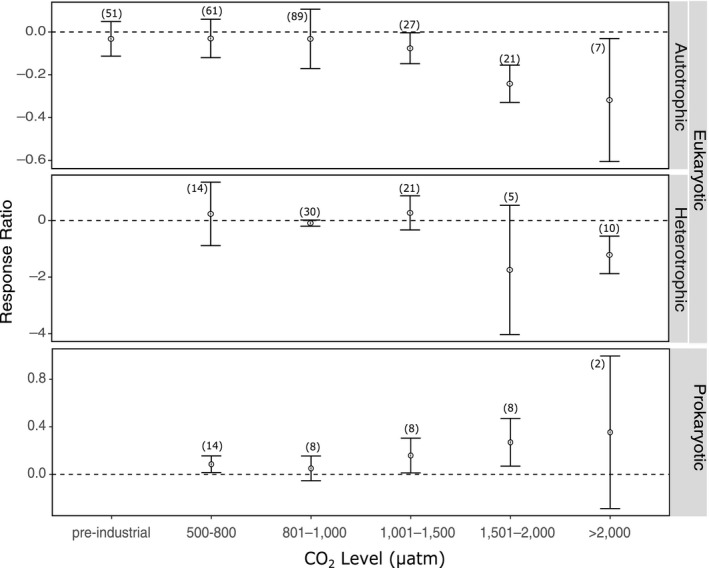
The effect of ocean acidification on heterotrophic and autotrophic eukaryotes, and prokaryotic organisms at different CO_2_ levels. Mean response ratios and 95% confidence intervals are shown, with the number of data points in each category given in brackets. A mean response ratio of zero (hashed line) indicates no effect

A total of 377 response ratios were calculated from the 60 studies included in the meta‐analysis (with a number of articles reporting results of multiple experiments and numerous biological responses). Of these response ratios, 40 were for prokaryotes (bacteria) and 337 for eukaryotic organisms. Of the eukaryotic response ratios, 257 were for autotrophic organisms (phytoplankton and macroalgae) and 80 for heterotrophic organisms (invertebrates and fish). Within these groups, there was significant heterogeneity (*p* < .001), indicating that variability between studies is greater than expected by chance alone (Tables S2–S6). Despite this, there are some clear trends in the response of Antarctic marine organisms to increased CO_2_ (Figure 2). Autotrophs showed a significant negative response to ocean acidification at CO_2_ levels >1,000 μatm, whereas in heterotrophs, the response is mainly negative above 1,500 μatm CO_2_. In contrast, the prokaryotes show an increasingly positive response with increasing CO_2_ (Figure 2).

**FIGURE 3:**
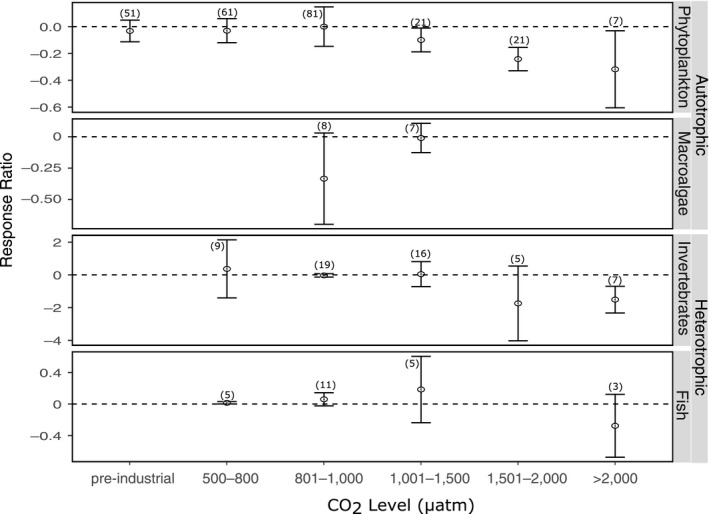
The effect of ocean acidification on phytoplankton, macroalgae, invertebrates, and fish at different CO_2_ levels. Mean response ratios and 95% confidence intervals are shown, with the number of data points in each category given in brackets. A mean response ratio of zero (hashed line) indicates no effect

The eukaryotic responses were subdivided to determine the effect of ocean acidification on phytoplankton, invertebrates, fish, and macroalgae (Figure 3). Significant heterogeneity was observed in each of these groups (Tables S3–S6). Nevertheless, responses to ocean acidification were significant in all groups. A total of 243 of the 257 autotrophic response ratios were for phytoplankton with only 3 studies conducted on macroalgae (from which 14 response ratios were calculated). For phytoplankton, including all the data and no a priori defined groups, there was significant heterogeneity between studies in their response to increased CO_2_ (*Q* = 221,529.7, *df* = 242, *p* < .001, Table S3). The response ratios showed an overall negative response to CO_2_ levels >1,000 μatm (Figure 3) but still significant heterogeneity at all CO_2_ levels (*p* < .0001, Table S3). For macroalgae, only 14 response ratios were calculated from three studies, with significant heterogeneity across the mean response of macroalgae both with all response ratios together, as well as when separated by CO_2_ level (Table S4). Despite this, the response in the 801–1,000 μatm CO_2_ category was mainly negative but the effect of CO_2_ in the 1,001–1,500 μatm level was less clear (Figure 3).

The response of heterotrophic eukaryotic organisms (invertebrates and fish) to CO_2_ was negative in CO_2_ treatments exceeding 1,500 μatm. For invertebrates, when these response ratios were combined, irrespective of the species studied or the response measured, their response to increased CO_2_ was mainly negative (Figure 3). Significant heterogeneity was observed within each CO_2_ category (*p* < .0001 for each CO_2_ category, Table S5). The response is less clear in fish, where the response ratios are based on a total of 8 studies from which 24 response ratios were calculated.

Groups of marine organisms differ greatly in their sensitivity to increased CO_2_ (Table 2). Bacteria are positively affected irrespective of the CO_2_ concentration. The few studies of fish also suggest that they are positively affected by CO_2_ concentrations <1,500 μatm and negatively affected >2,000 μatm, while their responses between 1,500 and 2,000 μatm are currently unknown. In contrast, phytoplankton, macroalgae, and invertebrates are all negatively affected. Invertebrates are the most sensitive to ocean acidification of the taxonomic groups examined, with a negative response to any increase in CO_2_ concentrations exceeding current levels in Antarctic waters (>500 μatm). Phytoplankton, although less sensitive, were negatively affected at CO_2_ levels above 1,000 μatm. The few studies on macroalgae provide some indication that they may be negatively affected, but the variation around the response is large and further data are required to gain a more conclusive response.

**TABLE 2:**
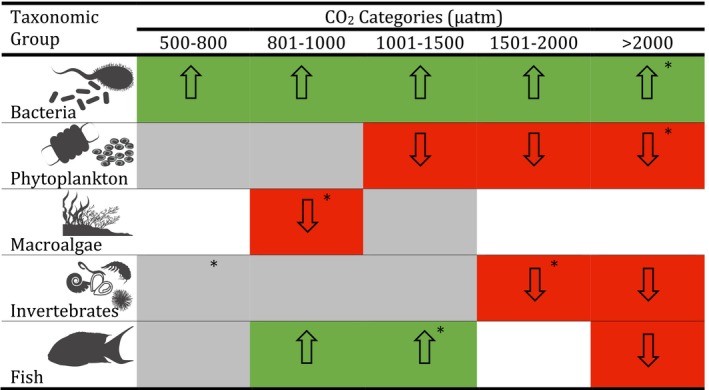
The effect of ocean acidification on bacteria, phytoplankton, macroalgae, invertebrates, and fish at different CO_2_ levels. Responses are either positive (green), negative (red), no effect (gray), or no data (blank). Responses based on few data points with consequently high variance are marked with *

**FIGURE 4:**
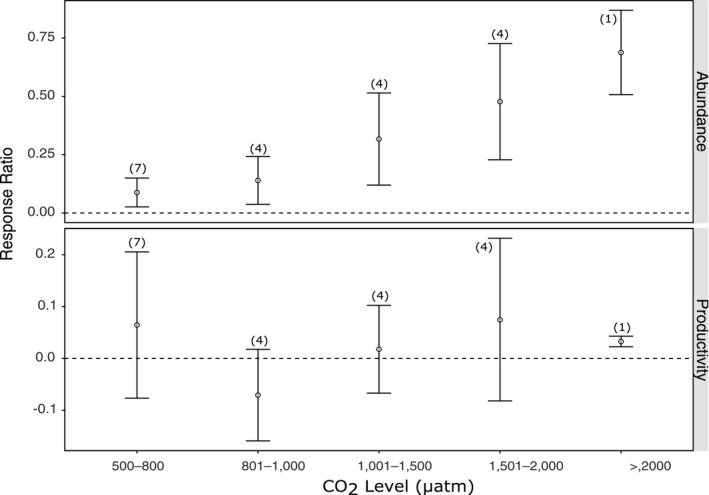
The effect of ocean acidification on the biological responses of bacteria at different CO_2_ levels. Mean response ratios and 95% confidence intervals are shown, with the number of data points in each category in brackets. A mean response ratio of zero (hashed line) indicates no effect

Few studies have been conducted on the effects of ocean acidification on bacteria in Antarctic and Southern Ocean waters; however, these few provide growing evidence that bacterial communities may also be affected. Four studies met the selection criteria for the meta‐analysis; Maas et al. (2013), Thomson, Davidson, and Maher (2016), Deppeler et al. (2018) and Westwood et al. (2018). These studies reported results that allowed 40 response ratios to be calculated. There is high heterogeneity in the response of bacteria to increased CO_2_ when these are combined, separated by CO_2_ level and/or biological response (*p* < .05, Table S2). Despite the significant heterogeneity, clear, positive response to increased CO_2_ was found for bacteria (Figure 2), particularly the increase in abundance with increasing CO_2_ (Figure 4). This trend is less clear for productivity (Figure 4).

### 3.1 Phytoplankton

**FIGURE 5:**
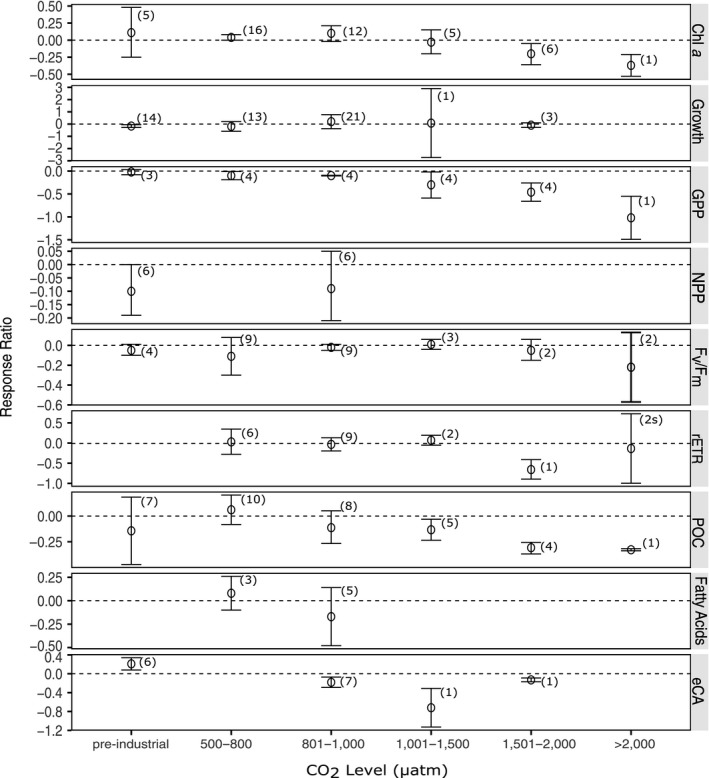
The effect of ocean acidification on the biological responses of phytoplankton to increased CO_2_ levels. Mean response ratios and 95% confidence intervals are shown, with the number of data points in each category in brackets. A mean response ratio of zero (hashed line) indicates no effect. Refer to Table 1 for abbreviation definitions

A range of biological responses have been used to quantify the effects of ocean acidification on phytoplankton with the nature and magnitude of the effect differing among biological responses (Figure 5). A negative effect was found on gross primary productivity (GPP), the activity of extracellular carbonic anhydrase (eCA), and the concentration of particulate organic carbon (POC) at CO_2_ levels above 500 μatm and above 801–1,000 μatm for POC concentration. The concentration of chlorophyll *a* was mainly positively affected by moderate increases in CO_2_ (≤1,000 μatm) but above this level the effect was increasingly negative. A clear response could not be discerned for growth rate, *F*_v_/*F*_m_ or rETR with variability in their response to altered CO_2_ concentrations. Although significant responses were found at a number of CO_2_ levels, there was still significant heterogeneity at all CO_2_ levels for both chlorophyll *a* concentration and growth rates. For example, the response ratio of growth rate at pre‐industrial levels is significantly negative (*p* = .0417) but there was also significant heterogeneity (*Q* = 90.511, *df* = 13, *p* < .001, Table S3), indicating that there is significant variation in the magnitude that growth rate was reduced in studies. The only biological responses in which the heterogeneity was not significant when separated by CO_2_ level were gross primary productivity at <1,000 μatm, *F*_v_/*F*_m_ at all levels except 500–800 and 801–1,000 μatm, POC concentration at 1501–2,000 μatm, fatty acid content at 500–800 μatm, and eCA activity at all levels. All other biological responses other CO_2_ levels had significant heterogeneity (Table S3).

**FIGURE 6:**
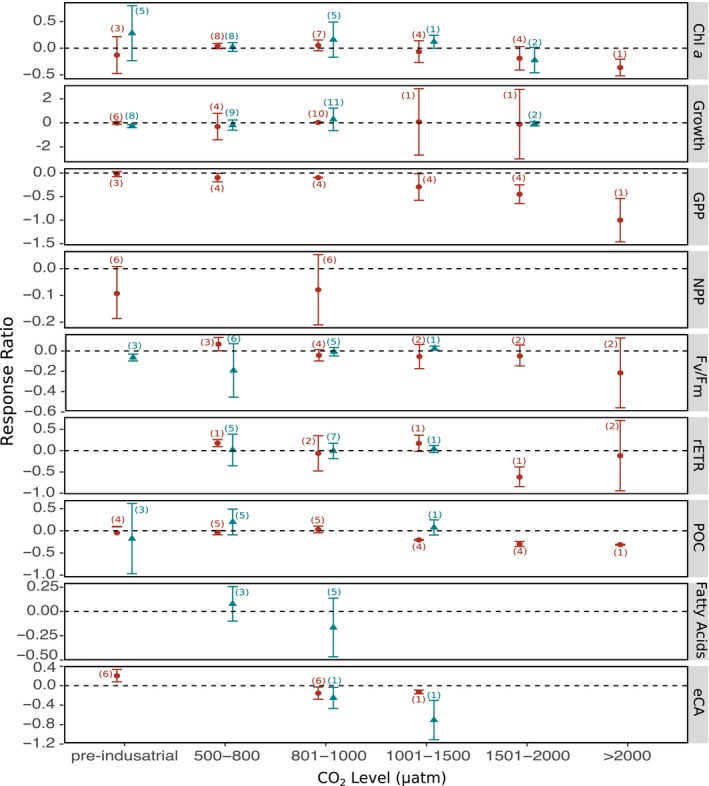
The effect of ocean acidification on the biological responses of phytoplankton to increased CO_2_ levels, separating studies on single species (shown in blue triangles) from community‐level studies (shown in red circles). Mean response ratios and 95% confidence intervals are shown, with the number of data points in each category in brackets. A mean response ratio of zero (hashed line) indicates no effect. Refer to Table 1 for abbreviation definitions

Responses of phytoplankton were separated depending on the experimental procedure comparing studies on a single species in culture to those performed on natural, mixed communities (Figure 6). Biological responses to ocean acidification differed between the two types of studies, and while often the results were highly variable, a summary table of thresholds levels of CO_2_ above which negative effects are observed showed that phytoplankton communities are more sensitive to increased CO_2_ than phytoplankton studied as a single species in a culture (Figure 6 and Table 3). For example, negative effects are observed in phytoplankton community *F*_v_/*F*_m_ and POC while the single‐species studies results did not show a clear response (Table 3). In addition, gross primary productivity of phytoplankton community was negatively affected at CO_2_ levels above 500 μatm, noting that this biological measurement is not in single‐species, culture studies (Table 3).

**TABLE 3:** Threshold levels of CO_2_ (μatm) for a range of biological responses for phytoplankton separating results of studies on single species from those at a community level. Responses are either negative (red), no effect (gray), or uncertain/no data (blank)

Biological response	Single species	Community
Chlorophyll *a*	1,500	1,500
Growth rate	No effect	No effect
Gross primary productivity		500
Net primary productivity		Few studies
*F*_v_/*F*_m_	No effect	Highly variable
rETR	No effect	Highly variable
POC concentration	No effect	1,000
Fatty acid content	High variable	
eCA activity	800	800

### 3.2 Macroalgae

**FIGURE 7:**
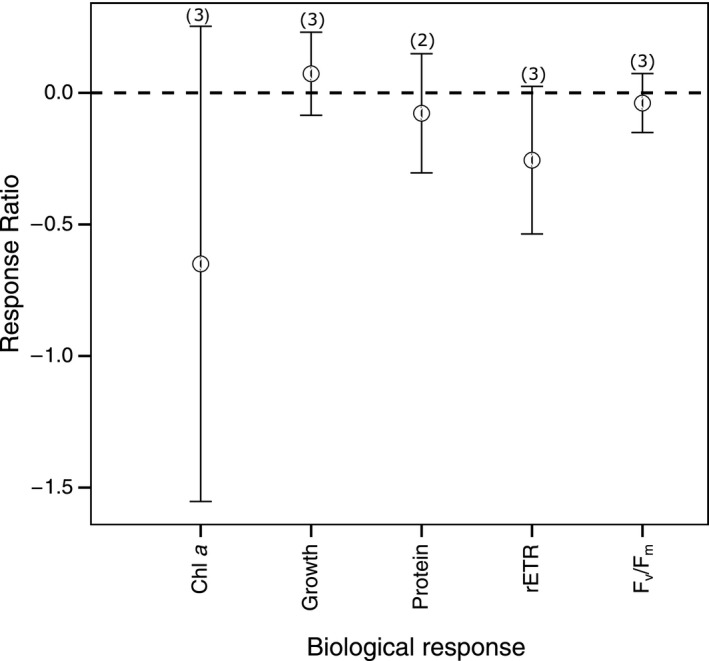
The effect of ocean acidification on the biological responses of macroalgae. Mean response ratios and 95% confidence intervals are shown, with the number of data points in each category in brackets. A mean response ratio of zero (hashed line) indicates no effect. Refer to Table 1 for abbreviation definitions

**FIGURE 8:**
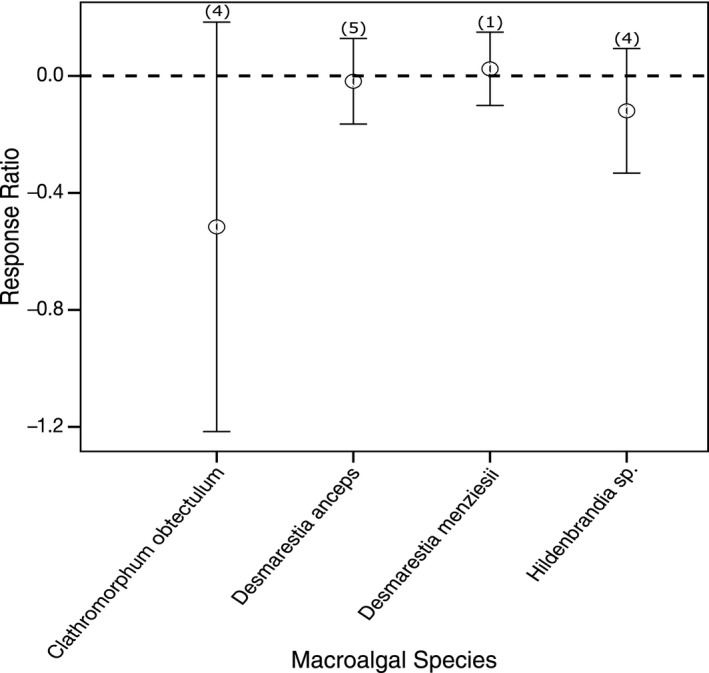
The effect of ocean acidification on species of macroalgae. Mean response ratios and 95% confidence intervals are shown, with the number of data points in each category in brackets. A mean response ratio of zero (hashed line) indicates no effect

Only three studies have investigated the effect of ocean acidification on macroalgae south of 60°S (Iniguez, Heinrich, Harms, & Gordillo, 2017; Schoenrock et al., 2016; Schram, Schoenrock, McClintock, Amsler, & Angus, 2017) and most of which employed CO_2_ treatment levels between 800 and 1,000 μatm. Due to the small data set available, all CO_2_ treatment levels were combined and data compared among biological responses (Figure 7) and species (Figure 8). While the heterogeneity is significant in the response of macroalgae to increased CO_2_ (Table S4), it appears that rETR is the most sensitive measure in macroalgae to increased CO_2_ (Figure 7). The concentration of chlorophyll *a* was also negatively affected by increased CO_2_, but there is high variance around this mean response. Comparing among species, heterogeneity was again high (Table S4). The crustose algal species, *Clathromorphyum obtectulum* and *Hildenbrandia* sp., appear to be more sensitive than fleshy species, *Desmarestia anceps* and *D. menzieseii* despite *C. obtectulum* and *Hildenbrandia *sp*.* being investigated at a lower CO_2_ level (801–1,000 μatm) than *D. anceps* and *D. menzieseii* (1,001–1,500 μatm).

### 3.3 Invertebrates

**FIGURE 9:**
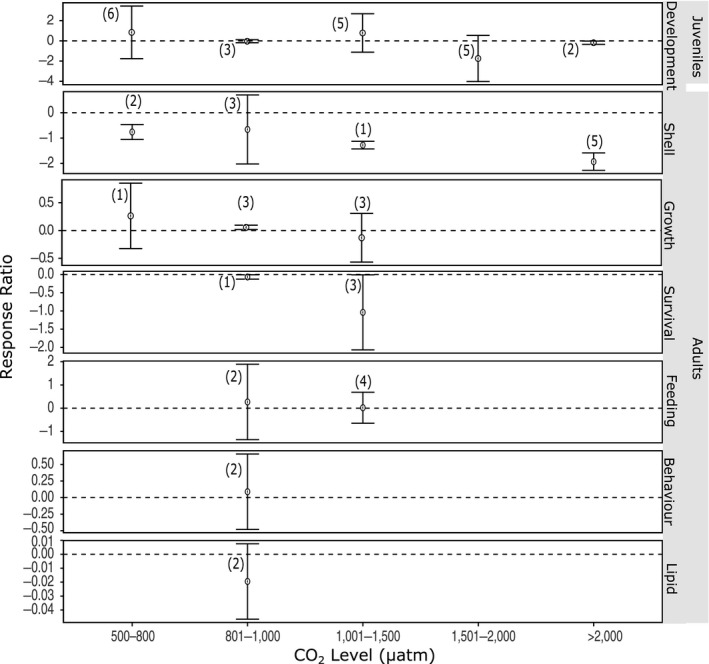
The effect of ocean acidification on the biological responses of invertebrates to increased CO_2_ levels. Mean response ratios and 95% confidence intervals are shown, with the number of data points in each category in level. A mean response ratio of zero (hashed line) indicates no effect. Refer to Table 1 for abbreviation definitions

For invertebrates, developmental stages, shell state, and survival of adults were found to be sensitive to increased CO_2_ (Figure 9). Larval life stages showed reduced normal development and fertilization rate with increasing CO_2_ >1,500 μatm. In adults, elevated CO_2_ reduced survival, calcium carbonate production rates, and increased rates of dissolution and shell damage in those species with calcium carbonate shells (Figure 9). Although based on few studies, other measurements such as growth rate, behavior, and feeding appeared to be unaffected by increased CO_2_ (Figure 9). However, there was significant heterogeneity in most biological responses when separated by CO_2_ level and with all CO_2_ levels pooled (Table S5). The only exceptions to this were growth rate and lipid/protein content (*Q* = 1.496, *df* = 5, *p* = .914 and *Q* = 0.416, *df* = 1, *p* = .519, respectively), but the lipid/protein results are derived from a single study only (Schram, Schoenrock, McClintock, Amsler, & Angus, 2016).

### 3.4 Fish

**FIGURE 10:**
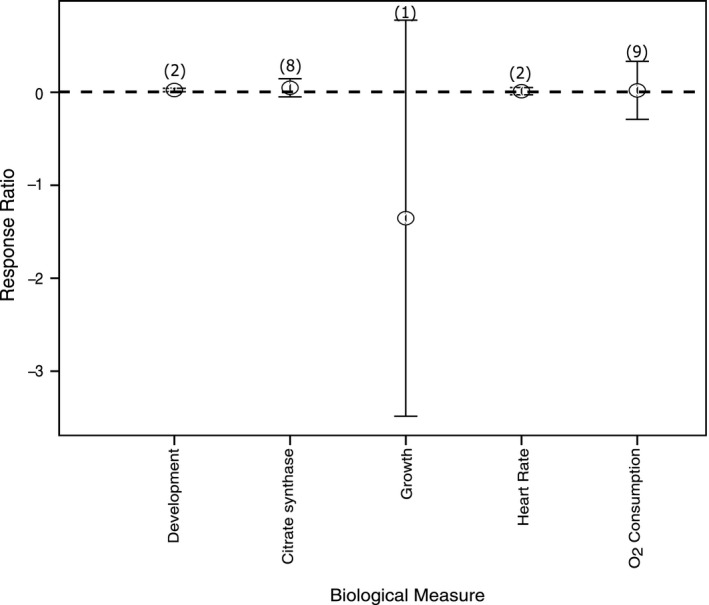
The effect of ocean acidification on the biological responses of fish. Mean response ratios and 95% confidence intervals are shown, with the number of data points in each category in brackets. A mean response ratio of zero (hashed line) indicates no effect. Refer to Table 1 for abbreviation definitions

The effect of ocean acidification on Antarctic and Southern Ocean fish has been little studied, with only 8 studies and a total of 24 responses available for this meta‐analysis (Davis et al., 2018; Davis, Miller, Flynn, & Todgham, 2016; Enzor, Hunter, & Place, 2017; Enzor, Zippay, & Place, 2013; Flynn, Bjelde, Miller, & Todgham, 2015; Strobel et al., 2012; Strobel, Graeve, Poertner, & Mark, 2013; Strobel, Leo, Pörtner, & Mark, 2013). While not ideal, due to the lack of data, all the CO_2_ treatment levels were combined and the results compared among biological responses, namely embryonic survival (development), cellular aerobic potential measured by citrate synthase enzyme activity in adults (CS), growth rate, heart rate, and oxygen (O_2_) consumption rate (Figure 10). There was no obvious difference in these among CO_2_ treatments (Figure 10). The cellular aerobic potential (CS) increases slightly under conditions of elevated CO_2_ while growth rate declines slightly but there was high variance surrounding all the biological responses; therefore, there is no certainty around this response.

## 4 DISCUSSION

This meta‐analysis shows that marine biota south of 60°S are vulnerable to ocean acidification, with only bacteria being positively affected by increases in CO_2_. Autotrophs, mainly phytoplankton, are negatively affected at CO_2_ levels above 1,000 μatm with decreases in chlorophyll *a*, gross primary productivity, and POC, as well as negative impacts on photosynthetic health (*F*_v_/*F*_m_ and rETR) ≥1,500 μatm (Figure 5). Invertebrates are particularly sensitive to increased CO_2_ with decreased survival, reduced calcification rate, and increased shell dissolution with any increase in CO_2_ above current levels (Figure 9). Early life invertebrate life stages are also vulnerable to ocean acidification with reduced fertilization rates and increased larval abnormalities found with increased CO_2_ levels (Figure 9). Few studies have been conducted on the effects of ocean acidification on fish in nearshore Antarctic and Southern Ocean waters; therefore, their response is uncertain. Despite some of the clear trends found in the response of bacteria, phytoplankton, and invertebrates to increased CO_2_, there is high variability in these trends between studies. This high variation is not surprising as there are relatively few studies investigating the effect of ocean acidification on marine biota in Antarctica (total of 48 studies), and those that have been conducted differed in the organisms studied, the biological responses measured, experimental design, and/or the location in which the studies were performed. Further studies are required to understand the most likely magnitude of response, and the affect this will have on the ecosystem services these marine organisms provide.

### 4.1 Bacteria

Ocean acidification may cause both direct and indirect effects on the bacterial communities. This meta‐analysis shows that increases in CO_2_ have an increasingly positive effect on heterotrophic bacterial abundance and productivity in Antarctic waters. Numerous studies in the Arctic have also reported a high resilience of bacteria to ocean acidification with either no effect or an increase in bacterial abundance, growth rate, and productivity with increases in CO_2_ (Allgaier, Riebesell, Vogt, Thyrhaug, & Grossart, 2008; Baragi, Khandeparker, & Anil, 2015; Grossart, Allgaier, Passow, & Riebesell, 2006; Paulino, Egge, & Larsen, 2007; Wang et al., 2016). Grossart et al. (2006) and Thomson et al. (2016) suggest that these changes in the bacterial community are indirect and driven by CO_2_‐induced changes in the phytoplankton community, to which they are tightly coupled. Of the four bacterial studies in this meta‐analysis, only Maas et al. (2013) reported biological responses other than abundance and productivity. Maas et al. (2013) found an increase in the activity of some extracellular enzymes as well as a shift in community composition with increased CO_2_. These trends have also been observed in high‐latitude Northern Hemisphere studies, including the PeECE III and EPOCA mesocosm experiments (Allgaier et al., 2008; Motegi et al., 2013; Paulino et al., 2007; Piontek et al., 2013; Roy et al., 2012; Sperling et al., 2013; Zhang et al., 2013). This suggests that while changes in abundance may be due to indirect CO_2_ effects, bacteria can still be directly affected by ocean acidification. There is some evidence globally that increases in CO_2_ can cause shifts in the bacterial community composition, with *Bacteriodetes*, *Polaribacter*, and free‐living bacteria are apparently more vulnerable to increased CO_2_ concentrations than other bacterial groups (Sperling et al., 2013; Zhang et al., 2013). Bacterial communities exposed to elevated CO_2_ also had a decrease in diversity and become dominated by CO_2_‐tolerant species (Maas et al., 2013). This suggests that bacterial communities may be quick to adapt to increases in CO_2_ by changing their community composition. However, many studies have also found no significant change in bacterial community composition with elevated CO_2_ and therefore, in many communities, bacterial diversity may be resilient to ocean acidification (Lin et al., 2018; Lindh et al., 2013; Oliver, Newbold, Whiteley, & Gast, 2014; Roy et al., 2012; Wang et al., 2016).

### 4.2 Phytoplankton

There is conflicting evidence in the literature on the sensitivity of noncalcifying phytoplankton to ocean acidification. This meta‐analysis shows that phytoplankton south of 60°S are negatively affected by increases in CO_2_ at levels above 1,000 μatm, but there is often a positive response below this threshold (Figure 5). Therefore, it is important that the CO_2_ treatment levels used in studies are taken into account when interpreting potential effects of increased CO_2_ and comparing effects between studies. The highest CO_2_ treatment commonly in ocean acidification experiments is approximately 800 μatm. This is below the threshold level of 1,000 μatm identified in the meta‐analysis in this study. Below 1,000 μatm, growth rate, chlorophyll *a* concentration, and the ratio of *F*_v_/*F*_m_ are either unaffected or significantly increased, but above growth rates, photosynthetic health (*F*_v_/*F*_m_ and rETR), and POC concentration are reduced (Figure 5). This threshold of 1,000 μatm is clearly shown in a series of experiments on nearshore, natural microbial communities in Prydz Bay, East Antarctica, where despite different starting communities and nutrient levels there was a consistent threshold for change in the microbial community structure and function above 1,000 μatm (Davidson et al., 2016; Deppeler et al., 2018; Hancock et al., 2018; Thomson et al., 2016; Westwood et al., 2018).

This meta‐analysis highlights the importance of studying marine organisms as part of a community or ecosystem, demonstrating that single‐species studies often underestimate the effects of ocean acidification as they fail to include complex interactions among species and trophic levels (Figure 6). Single‐species studies also miss potential shifts in the community composition, as commonly found in phytoplankton studies. A number of phytoplankton studies south of 60°S have found a significant shift in community composition; however, there is disagreement on the direction of community change. Some studies found an increase in larger diatoms (Feng et al., 2010; Hoppe et al., 2013; Maas et al., 2013; Tortell et al., 2008), while other studies found that smaller cells were promoted at higher CO_2_ levels (Davidson et al., 2016; Hancock et al., 2018; Thomson et al., 2016). This may be partially due to regional differences in phytoplankton communities among these studies but again also reflects the CO_2_ levels used relative to the threshold concentration that elicits community change. Hancock et al. (2018) found that larger diatom species were promoted at intermediate CO_2_ concentrations (500–1,000 μatm), but above 1,000 μatm, there was a significant decline in larger diatoms and a shift to a community dominated by smaller cells. This observation could explain some of the variability in results of the other studies, where 750–800 μatm is commonly the highest CO_2_ treatment tested (i.e., Tortell et al., 2008, Feng et al., 2010, Hoppe et al., 2013 and Young, Kranz, Goldman, Tortell, & Morel, 2015). Studies by Tortell et al. (2008), Feng et al. (2010), Hoppe et al. (2013) and Hancock et al. (2018) all show that larger diatoms are promoted by increases in CO_2_ up to 1,000 μatm. Thus, care must be taken when comparing the effects of ocean acidification among studies with different CO_2_ treatment levels.

A number of the biological responses of phytoplankton to increased CO_2_ had a parabolic trend, with positive responses with moderate increases in CO_2_ but above 1,000 μatm an increasingly negative response (i.e., chlorophyll *a* concentration and growth rate, Figure 5). A possible explanation for the parabolic responses to increased CO_2_ could be the interaction of the beneficial effects of CO_2_ with the inhibitory effects of the coincident rise in H^+^ ions. Current levels of CO_2_ in ambient seawater are limiting to phytoplankton growth due to the low affinity of the enzyme ribulose‐1,5‐bisphosphate carboxylase/oxygenase (RuBisCo) for CO_2_ (Reinfelder, 2011). This enzyme is involved in the first major step of carbon fixation and is present in all eukaryotic phytoplankton. Its half‐saturation concentration for CO_2(aq) _is, however, substantially higher than present day naturally occurring CO_2_ levels in seawater (Reinfelder, 2011). To overcome this low affinity of RuBisCo, phytoplankton have developed carbon concentration mechanisms (CCMs) so high rates of photosynthesis can be maintained, but this is an energy‐consuming process (Raven, Cockell, & Rocha, 2008). At elevated CO_2_, cells possessing efficient CCMs can downregulate these carbon concentrating mechanisms, thereby saving energy while sustaining photosynthetic rate (Rost, Zondervan, & Wolf‐Gladrow, 2008). This is supported by the findings of this meta‐analysis where the activity of extracellular carbonic anhydrase (eCA), an enzyme involved in carbon acquisition and an indicator for CCM activity, is increased at CO_2_ levels below ambient, but decreased above ambient, reflecting a downregulation of CCMs at increased CO_2_. If the CCMs are downregulated, then photosynthesis and growth would increase due to the energy saved from not having to operate the CCMs. However, at CO_2_ levels above 1,000 μatm growth rate, chlorophyll *a* concentration, primary productivity (NPP and GPP), and photosynthetic health declined. It is likely that the coincident rise in H^+^ ions with increased CO_2_ is resulting in the requirement for the phytoplankton to expend energy on proton pumps to maintain intracellular pH (Cyronak, Schulz, & Jokiel, 2015; Deppeler et al., 2018; Gafar, Eyre, & Schulz, 2017; McMinn, Müller, Martin, & Ryan, 2014; Taylor, Brownlee, & Wheeler, 2012). The use of CCMs in phytoplankton varies between species, and some studies have found that diatoms and *Phaeocystis* are capable of indirect or direct HCO_3_^‐^ uptake and are not limited by CO_2_ (Cassar, Laws, Bidigare, & Popp, 2004; Goldman, 1999; Martin & Tortell, 2006; Tortell, DiTullio, Sigman, & Morel, 2002; Tortell & Morel, 2002; Tortell, Reinfelder, & Morel, 1997). For these larger species, the intolerance to increased CO_2_ is likely to reflect their need to operate proton pumps to combat the rise in H^+^ concentration.

The effect of ocean acidification on sea‐ice algae and in long‐term phytoplankton experiments has not been included in this meta‐analysis as they are difficult to compare to short‐term pelagic phytoplankton studies. Sea‐ice algae experience large seasonal fluctuations in pH and ambient CO_2_ levels can exceed >1,000 μatm (Coad, McMinn, Nomura, & Martin, 2016; McMinn et al., 2014, 2017; Miller et al., 2011). McMinn (2017) provides a review on the effect of ocean acidification on sea‐ice microbial communities and concludes that sea‐ice brine algal communities tolerate elevated levels of CO_2_ with negative effects on growth and photosynthesis only at the most extreme CO_2_ levels, >2,500 μatm. This contrasts with the tolerance of pelagic phytoplankton which were negatively affected at CO_2_ levels above 1,000 μatm. Also not included in the meta‐analysis are data from long‐term studies, such as those by Trimborn et al. (2014) and Torstensson et al. (2015). These studies aim to investigate the acclimation capacity of phytoplankton to increased CO_2_, as opposed to examining the impacts of exposure to increased CO_2_ on key biological responses. Torstensson et al. (2015) found only a small reduction in growth of *Nitzschia lecointei* after 147 days at 960 μatm (with the experiment run for 194 days in total), although Trimborn et al. (2014) highlight that responses can be species‐specific at 1,000 μatm, which is a similar tolerance level found in this meta‐analysis. Studies on the ability of phytoplankton to acclimate and/or adapt to increased CO_2_ are rare. The few studies conducted globally suggest that phytoplankton could adapt, partially or totally negating the negative effects of increased CO_2_ over the longer time scales at which the oceans are actually changing (i.e., Lohbeck, Riebesell, & Reusch, 2012).

### 4.3 Macroalgae

Very few studies have been conducted on macroalgae in Antarctic waters. From the few studies available, it appears crustose algal species are more vulnerable than noncalcifying fleshy species to ocean acidification, but with species‐specific differences (Figures 7 and 8). However, there is high uncertainty around this response and further studies are needed to accurately predict the response of macroalgae to ocean acidification in Antarctic waters.

Globally, crustose algae have been found to be sensitive to increases in CO_2_ with decreases in calcification, increased thalli dissolution, and decreased structural integrity (Agostini et al., 2018; Fabricius, Kluibenschedl, Harrington, Noonan, & De'ath, G., 2015; Hofmann & Bischof, 2014; Johnson & Carpenter, 2012; Kuffner, Andersson, Jokiel, Rodgers, & MacKenzie, 2008; McCoy & Kamenos, 2015; Roleda & Hurd, 2012). Ocean acidification reduces biogenic calcification by decreasing the calcium carbonate saturation resulting in reduced calcification and increased dissolution of crustose algal thalli. This reduces the structural integrity and protective function of crustose algae making them susceptible to bioerosion and increased grazing (Johnson & Carpenter, 2012; Koch, Bowes, Ross, & Zhang, 2013). In the west Antarctic Peninsula, the coverage of crustose algae can reach 77% in some subtidal benthic habitats but unfortunately little is known about the role of crustose algae in Antarctic benthic ecology (Amsler, Rowley, Laur, Quetin, & Ross, 1995; Schoenrock et al., 2016). In Arctic analogues, crustose algae provide an important habitat for benthic invertebrates and fish and play a key role in substrate stabilization of subtidal habitats (Chenelot, Jewett, & Hoberg, 2011; Teichert & Freiwald, 2014). Schoenrock et al. (2016) suggest that future ocean acidification conditions could alter algal community structure due to the differences in CO_2_ tolerance among the species. A change in the algal community structure and a decline in crustose algal integrity could have significant flow‐on effects to the Antarctic benthic ecosystem, particularly benthic communities which are closely associated with or are dependent on macroalgae (Peck, 2018; Schoenrock et al., 2016).

### 4.4 Invertebrates

This meta‐analysis shows that Antarctic invertebrates are particularly sensitive to ocean acidification with negative effects on calcification, reproduction, and survival. This agrees with the findings of Kroeker et al. (2010, 2013) but globally the sensitivity of invertebrates to elevated CO_2_ is phylum‐specific, with echinoderms and mollusks less tolerant than crustaceans (Kroeker et al., 2010, 2013). The limited number of studies conducted on Antarctic invertebrates, and the wide range of biological responses measured, prevented phylum‐specific conclusions from being determined in the current meta‐analysis. Despite this, it is clear that invertebrates could be threatened at elevated CO_2_ concentrations with reduced fertilization rates, increased larval abnormalities, decreased calcification, and increased shell dissolution (Figure 9).

Calcifying organisms are thought to be indicator organisms for ocean acidification as they are at heightened risk from increasing ocean acidification due to the reduction in calcium carbonate saturation state (Fabry et al., 2009; McNeil & Matear, 2008; Orr et al., 2005). Decreases in the saturation state of calcium carbonate due to ocean acidification result in decreased calcification and increased dissolution of calcium carbonate shells, with some invertebrate taxa more vulnerable than others (Andersson, Mackenzie, & Gattuso, 2011). Pteropods, for example, are reportedly particularly vulnerable to ocean acidification due to their weaker aragonite shells (Bednarsek, Tarling, Bakker, Fielding, & Feely, 2014; Gardner, Manno, Bakker, Peck, & Tarling, 2018). In addition, transcriptomic studies suggest that pteropods may not have the plasticity required to adapt to ocean acidification over the timescale in which they will be exposed to undersaturated conditions (Johnson & Hofmann, 2017). For Antarctic echinoderms, distributions show that species living at depth below the current calcite saturation horizon have thinner, more weakly calcified shells than those living above this horizon (Sewell & Hofmann, 2011). This response appears to be species‐specific as some Antarctic echinoderms show no clear trend in calcification with depth or the proportion of highly soluble magnesium‐calcite in their skeletons, suggesting their calcification could depend on the acid–base status of the species (Collard, Ridder, David, Dehairs, & Dubois, 2015; Duquette, Halanych, Angus, & McClintock, 2018). Furthermore, McClintock et al. (2009) showed that the composition of the invertebrate's shell (calcite or aragonite) did not affect the vulnerability of those shells to a decrease in the saturation state of calcium carbonate. Rather, the vulnerability of shells to ocean acidification depended on the strength of the shell matrix (Harper, 2000; McClintock et al., 2009). However, the experiments conducted by McClintock et al. (2009) were conducted on empty shells of Antarctic invertebrates. Cyronak et al. (2015) show that the vulnerability of calcifying organisms to ocean acidification is not due a decline in calcium carbonate saturation driving a decrease in calcification rates, but rather the increase in H^+^ ions associated with the drop in pH, which makes it difficult for organisms to maintain their pH homeostasis.

Early developmental stages of invertebrates are known to be more sensitive to changes in their environment as their regulatory mechanisms are still developing (Burggren & Warburton, 2005; Kurihara, 2008). This trend in sensitivity is consistent with the current study, which showed negative effects of CO_2_ concentration on invertebrate development above 1,500 μatm, including decreased rates of fertilization, egg cleavage and hatching, decreased survival of larvae, increased abnormalities, and delayed development of later larval stages (Bylenga, Cummings, & Ryan, 2015, 2017; Byrne et al., 2013; Ericson et al., 2012; Ericson, Lamare, Morley, & Barker, 2010; Foo et al., 2016; Gonzalez‐Bernat, Lamare, & Barker, 2013; Karelitz et al., 2016; Kawaguchi et al., 2010, 2013). This contrasts with biological responses of adults, which only showed a negative response in shell state and survival, although comparatively more studies have focused on the effects of ocean acidification on juveniles than adult invertebrates (Figure 9). All the species included in this meta‐analysis are free spawners with planktonic larvae (Ansell & Harvey, 1997; Nicol, 2006; Pearse & Bosch, 1986; Quetin & Ross, 1984). Waters below 100 m can have CO_2_ levels considerably higher than at the surface (Kawaguchi et al., 2010, 2013). This means larvae and eggs could experience higher CO_2_ conditions in mid‐pelagic waters earlier than predicted for surface waters (Kawaguchi et al., 2010). The bivalve *L. elliptica* may be particularly vulnerable as it uses aragonite to form its shell and spawns during the winter (Ansell & Harvey, 1997) when seawater pH is at its lowest and aragonite is predicted to become seasonally undersaturated by 2030 (Gibson & Trull, 1999; McNeil & Matear, 2008; Roden et al., 2013). What remains unclear is whether marine invertebrates will adapt to reduced pH, and whether individuals that survive the larval phase and grow to adulthood will have stronger, more resilient offspring. Suckling et al. (2015) showed that *S. nemayeri* grown at 900–1,400 μatm CO_2_ acclimate both their metabolic and reproductive physiology, and after 2 years, there was no significant difference in urchins exposed to current day and elevated CO_2_ conditions. Ericson et al. (2018) also showed Antarctic krill, *Euphausia superba*, have a high resilience to elevated CO_2_ when exposed over an entire year. These and other studies suggest that some invertebrates may be able to adapt over time to ocean acidification conditions and produce more resilient offspring, but further long‐term studies are needed to fully explore this intergenerational adaptive capacity (Peck, 2018).

As invertebrates play an important role in the Antarctic ecosystem, any decline in their abundance or change in community composition due to ocean acidification could have significant implications for the Antarctic and Southern Ocean ecosystem and the food webs they support. For example, pteropods are a major food source for many zooplankton, fish, and higher predators (Falk‐Petersen, Sargent, Kwasniewski, Gulliksen, & Millar, 2001; Hopkins, 1987) and play a role in the export of carbon to the deep ocean (Manno, Tirelli, Accornero, & Fonda Umani, 2010). Invertebrates such as sea stars, sea urchins, limpets, sea snails, bivalves, and brachiopods are important components in benthic communities as bioturbators, major grazers on macroalgae, and benthic diatoms and play a key role in energy transfer in the Antarctic benthos (McClintock, 1994; Widdicombe, Austen, Kendall, Warwick, & Jones, 2000).

### 4.5 Fish

Fish appear to tolerate ocean acidification but this is based on only a few studies on the effect of altered carbonate chemistry, highlighting the need for more investigations and on these and other higher trophic level organisms. Global meta‐analyses on the effect of ocean acidification on fish found no effect or only a slight positive effect of increased CO_2_ on fish growth, but again from limited data (Kroeker et al., 2010, 2013). Some studies suggest that fish may expend more energy under elevated CO_2_ conditions to maintain osmoregulation particularly when exposed for longer periods of increased CO_2_ (Flynn et al., 2015; Kidder, Petersen, & Preston, 2006). Studies conducted on the capacity of Antarctic notothenioids have found that some species (*Trematomus newnesi* and *Notothenia rossi*) have little ability to physiologically adjust to environmental change (Enzor et al., 2013; Strobel, Graeve, et al., 2013), while others (*Pagothenia borchgrevinki*, *T. bernacchii* and *T. hansoni*) rapidly acclimate (Enzor et al., 2013; Huth & Place, 2016). These may be species‐specific differences in the tolerance of ocean acidification that favor the increasing dominance of some taxa in the future. Unfortunately, the present lack of studies reporting the effects of ocean acidification on fish both in Antarctic and globally means the response of fish is unclear and highlights the need for further investigations.

### 4.6 Conclusions and future directions

This meta‐analysis highlights the sensitivity of marine organisms in the Southern Ocean and nearshore Antarctic waters to future ocean acidification conditions. The threshold level for negative responses is ≈1,000 μatm for autotrophs and ≈500 μatm for heterotrophs, with only prokaryotes (bacteria) being positively affected by increased levels of CO_2_. By year 2100, when nearshore Antarctic waters are projected to have CO_2_ levels between 880 and 1,000 μatm (under the IPCC business as usual scenario, RCP8.5), these waters could experience an increase in the abundance of bacteria, reduction in primary productivity, and phytoplankton communities could be become dominated by smaller cells. Invertebrates could also be threatened with reductions in fertilization rate and increased larval abnormalities occurring at a CO_2_ concentrations above 1,500 μatm. Adult invertebrates may struggle to both make and maintain their shells due to the negative effects on calcification and increased shell dissolution with undersaturation of aragonite predicted for some areas of the Southern Ocean by 2100 (McNeil & Matear, 2008).

There have been an increasing number of studies focusing on whole ecosystem responses, which take into account natural variability in CO_2_ levels into the experimental design. Nearshore Antarctic waters, where the majority of the studies included in this meta‐analysis were conducted, have large annual fluctuations in CO_2_ due to changes in sea‐ice coverage and primary production (Gibson & Trull, 1999; McNeil & Matear, 2008; Roden et al., 2013). Kapsenberg et al. (2015) argue that Antarctic Ocean acidification experiments should incorporate projected pH changes as well as natural temporal pH variability due to the influence these temporal changes could have on organism's ability to adapt to future acidification conditions. Superimposing seasonal pH variability on projected future pH levels has been shown to increase the amplitude of seasonal changes in pH (Kapsenberg et al., 2015). This could be exacerbated further with the reduction in primary productivity found in this meta‐analysis which would further decrease pH and aragonite undersaturation in the future (Kapsenberg et al., 2015). Other than in microbial studies, the majority of ocean acidification studies on Antarctic marine biota have been conducted on a single species in isolation. Such studies are of limited predictive value as they do not include the complex interactions among species and trophic levels that occur in nature. Differences in single‐species versus natural communities are demonstrated by the observed increase in sensitivity of phytoplankton communities to ocean acidification compared to monospecific studies described here. This highlights the need for community‐ and ecosystem‐level studies to better predict the effects of ocean acidification on the nearshore Antarctic and Southern Ocean ecosystem, such as the recent antFOCE experiment (Stark et al., 2018).

Ocean acidification is not occurring in isolation and changing ocean conditions including increasing temperatures are happening over entire ecosystems and longer time periods than most experiments cover. The Southern Ocean and nearshore Antarctic waters are also warming, sea‐ice is predicted to decline, winds are strengthening, upwelling and nutrient availability is changing, mixed layer depths are shallowing, and the ocean fronts are moving southward (Deppeler & Davidson, 2017; Stark, Raymond, Deppeler, & Morrison, 2019). These stressors interact, resulting in additive, synergistic, or antagonistic effects on organisms (Boyd, Brown, Schrum, & Krishnakumar, 2015). To better predict the potential response of organisms and ecosystems to global change, we need to develop an understanding of how organisms will cope when exposed to multiple stressors, plus understand the response of organisms to these stressors over longer time scales, during which organisms have time to acclimate and/or adapt to the changing environment.

This meta‐analysis shows that ocean acidification can detrimentally affect marine organisms south of 60°S and the ecosystem services they provide. There is currently poor spatial coverage, with most studies conducted in nearshore Antarctic waters surrounding the Antarctic Peninsula, the Ross Sea and Prydz Bay, East Antarctica where many Antarctic bases are located. While this lack of coverage and focus around the location of Antarctic bases is understandable given the logistical constraints of conducting Antarctic fieldwork and experiments, this limitation does need to be recognized when assessing the vulnerability of Antarctic marine organisms to ocean acidification. There is an overall lack of data available on the effect of ocean acidification on Antarctic marine organisms. Consequently, estimates of the effect of elevated CO_2_ in this meta‐analysis are highly variable, with most studies examining differing organisms and biological responses at different locations to all other studies. Outside of microbial studies, no studies have been conducted on natural communities or entire ecosystems (although see Stark et al., 2018), which could have quite different responses and sensitivities to monospecific studies. Better predictions of the future state of the Southern Ocean and nearshore Antarctic ecosystems require more studies that incorporate entire communities over timescales that allow organisms to acclimate and potentially adapt, to a range of CO_2_ and multistressor scenarios.

## CONFLICT OF INTEREST

None declared.

## AUTHOR CONTRIBUTION

Alyce Meredith Hancock: Conceptualization (lead); Data curation (lead); Formal analysis (lead); Investigation (lead); Methodology (lead); Project administration (lead); Software (lead); Validation (lead); Visualization (lead); Writing‐original draft (lead); Writing‐review & editing (lead). Catherine King: Conceptualization (supporting); Data curation (supporting); Investigation (supporting); Project administration (supporting); Supervision (supporting); Writing‐review & editing (supporting). Jonathan Stark: Conceptualization (supporting); Data curation (supporting); Funding acquisition (equal); Investigation (supporting); Supervision (supporting); Writing‐review & editing (supporting). Andrew McMinn: Data curation (supporting); Funding acquisition (equal); Investigation (supporting); Supervision (supporting); Writing‐review & editing (supporting). Andrew Davison: Conceptualization (supporting); Data curation (supporting); Funding acquisition (equal); Investigation (supporting); Supervision (supporting); Writing‐review & editing (supporting).

## Supporting information

Supplementary Material

## Data Availability

Raw data used in the meta‐analysis are available via the Australian Antarctic Division Data Centre, https://doi.org/10.26179/5e4f475eb6fbd.
