# Supplementary material for: Effects of ocean acidification on Antarctic marine organisms: A meta‐analysis
Source: Ecol Evol. 2020 Apr 16;10(10):4495–514. doi: 10.1002/ece3.6205 (PMC7246202; doi:10.1002/ece3.6205)
Supplement: Supplementary file 1 — Supplementary Material [file ECE3-10-4495-s001.pdf]

# Supplementary Material

# Effects of ocean acidification on Antarctic marine organisms: a meta-analysis

Table S1: Database search strategy with main themes (top line) and synonyms below. Searches were conducted using ‘and’ between columns and ‘or’ between rows for each column e.g. ocean or marine and acid* or pH and “Southern Ocean”. For Scopus database “ ” were replaced with { }.

| | | |
|---|---|---|
| ocean | acid* | “Southern Ocean” |
| marine | pH | Antarctic* |
| pelagic | ?CO2 | |
| coast* | DIC | |
| nearshore | “carbon dioxide” | |
| | “dissolved inorganic carbonate chemistry” | |
| | “carbonate chemistry” | |

**Supplementary Document 1: References for papers included in the meta-analysis**

Bednaršek, N., Tarling, G. A., Bakker, D. C. E., Fielding, S., and Feely, R. A. (2014). Dissolution dominating calcification process in polar pteropods close to the point of aragonite undersaturation. *PLoS ONE*, 9(10):1–14.

Black, J. G, Stark, J. S., Johnstone, G. J., McMinn, A., Boyd, P., McKinlay, J., Wootherspoon, S., and Runcie, J. W. (2019). *In situ* behavioural and physiological responses of Antarctic microphytobenthos to ocean acidification. *Scientific Reports*, 9: 1890.

Bylenga, C. H., Cummings, V. J., and Ryan, K. G. (2015). Fertilisation and larval development in an Antarctic bivalve, *Laternula elliptica*, under reduced pH and elevated temperatures. *Marine Ecology Progress Series*, 536:187–201.

Bylenga, C. H., Cummings, V. J., and Ryan, K. G. (2017). High resolution microscopy reveals significant impacts of ocean acidification and warming on larval shell development in *Laternula elliptica*. *PLoS ONE*, 12(4).

Byrne, M., Ho, M. A., Koleits, L., Price, C., King, C. K., Virtue, P., Tilbrook, B., and Lamarek, M. (2013). Vulnerability of the calcifying larval stage of the Antarctic sea urchin *Sterechinus neumayeri* to near-future ocean acidification and warming. *Global Change Biology*, 19(7):2264–2275.

Clark, D., Lamare, M., and Barker, M. (2009). Response of sea urchin pluteus larvae (Echinodermata: Echinoidea) to reduced seawater pH: a comparison among a tropical, temperature, and a polar species. *Marine Biology*, 156:1125-1137.

Coad, T., McMinn, A., Nomura, D., and Martin, A. (2016). Effect of elevated $CO_2$ concentration on microalgal communities in Antarctic pack ice. *Deep-Sea Research Part II*, 131:160–169.

Cross, E. L., Peck, L. S., and Harper, E. M. (2015). Ocean acidification does not impact shell growth or repair of the Antarctic brachiopod *Liothyrella uva* (Broderip, 1833). *Journal of Experimental Marine Biology and Ecology*, 462:29–35.

Davidson, A. T., McKinlay, J., Westwood, K., Thomson, P. G., van den Enden, R., de Salas, M., Wright, S., Johnson, R., and Berry, K. (2016). Enhanced $CO_2$ concentrations change the structure of Antarctic marine microbial communities. *Marine Ecology Progress Series*, 552:93–113.

Davis, B. E., Flynn, N. A., Miller, N. A., Nelson, F. A., Fangue, N. A., and Todgham, A. E. (2018). Antarctic emerald rockcod have the capacity to compensate for warming when uncoupled from $CO_2$-acidification. *Global Change Biology*, 24: e655-e670

Davis, B. E., Miller, N. A., Flynn, E. E., and Todgham, A. E. (2016). Juvenile Antarctic rockcod (*Trematomus bernacchii*) are physiologically robust to $CO_2$-acidified seawater. *Journal of Experimental Biology*, 219(8):1203–1213.

Deppeler, S., Petrou, K., Schulz, K. G., Westwood, K., Pearce, I., McKinlay, J., and Davidson, A. (2018). Ocean acidification of a coastal Antarctic marine microbial community reveals a critical threshold for $CO_2$ tolerance in phytoplankton productivity. *Biogeosciences*, 15(1):209.

Enzor, L. A., Hunter, E. M., and Place, S. P. (2017). The effects of elevated temperature and ocean acidification on the metabolic pathways of notothenioid fish. *Conservation Physiology*, 5.

Enzor, L. A., Zippay, M. L., and Place, S. P. (2013). High latitude fish in a high $CO_2$ world: Synergistic effects of elevated temperature and carbon dioxide on the metabolic rates of Antarctic notothenioids. *Comparative Biochemistry and Physiology, Part A*, 164(1):154–161.

Ericson, J. A., Ho, M. A., Miskelly, A., King, C. K., Virtue, P., Tilbrook, B., and Byrne, M. (2012). Combined effects of two ocean change stressors, warming and acidification, on fertilization and early development of the Antarctic echinoid *Sterechinus neumayeri*. *Polar Biology*, 35(7):1027–1034.

Ericson, J. A., Lamare, M. D., Morley, S. A., and Barker, M. F. (2010). The response of two ecologically important Antarctic invertebrates (*Sterechinus neumayeri* and *Parborlasia corrugatus*) to reduced seawater pH: effects on fertilisation and embryonic development. *Marine Biology*, 157(12):2689–2702.

Feng, Y., Hare, C. E., Rose, J. M., Handy, S. M., DiTullio, G. R., Lee, P. A., Smith, W. O., Peloquin, J., Tozzi, S., Sun, J., Zhang, Y., Dunbar, R. B., Long, M. C., Sohst, B., Lohan, M., and Hutchins, D. A. (2010). Interactive effects of iron, irradiance and $CO_2$ on Ross Sea phytoplankton. *Deep-Sea Research Part I Oceanographic Research Papers*, 57(3):368–383.

Flynn, E. E., Bjelde, B. E., Miller, N. A., and Todgham, A. E. (2015). Ocean acidification exerts negative effects during warming conditions in a developing Antarctic fish. *Conservation Physiology*, 3.

Foo, S. A., Sparks, K. M., Uthicke, S., Karelitz, S., Barker, M., Byrne, M., and Lamare, M. (2016). Contributions of genetic and environmental variance in early development of the Antarctic sea urchin *Sterechinus neumayeri* in response to increased ocean temperature and acidification. *Marine Biology*, 163(6).

Gonzalez-Bernat, M. J., Lamare, M., and Barker, M. (2013). Effects of reduced seawater pH on fertilisation, embryogenesis and larval development in the Antarctic seastar *Odontaster validus*. *Polar Biology*, 36(2):235–247.

Hancock, A. M., Davidson, A. T., McKinlay, J., McMinn, A., Schulz, K. G., and van den Enden, R. L. (2018). Ocean acidification changes the structure of an Antarctic coastal protistan community. *Biogeosciences*, 15.

Heiden, J P., Völkner, C., Jones, E. M., van de Poll, W., H., Buma, A. G. J., Meredith, M. P., de Baar, H. J. W., Bischof, K., Wolf-Gladrow, D., and Trimborn, S. (2019). Impact of ocean acidification and high solar radiation on productivity and species composition of a late summer phytoplankton community of the coastal Western Antarctic Peninsula. *Limnology and Oceanography*, 9999: 1-21.

Heiden, J. P., Thoms, S., Bischof, K., and Trimborn, S. (2018). Ocean acidification stimulates particulate organic carbon accumulation in two Antarctic diatom species under moderate

and high nature solar radiation. *Jounral of Phycology*, 54: 505-517.

Heiden, J. P., Bischof, K., and Trimborn, S. (2016). Light intensity modulates the response of two Antarctic diatom species to ocean acidification. *Frontiers in Marine Science*, 3:260.

Ho, M. A., Price, C., King, C. K., Virtue, P., and Byrne, M. (2013). Effects of ocean warming and acidification on fertilization in the Antarctic echinoid *Sterechinus neumayeri* across a range of sperm concentrations. *Marine Environmental Research*, 90:136–141.

Hoogstraten, A., Timmermans, K. R., and de Baar, H. J. W. (2012). Morphological and physiological effects in *Proboscia alata* (Bacillariophyceae) grown under different light and $CO_2$ conditions of the modern Southern Ocean. *Journal of Phycology*, 48(3):559– 568.

Hoshijima, U., Wong, J. M., and Hofmann, G. E. (2017). Additive effects of $pCO_2$ and temperature on respiration rates of the Antarctic pteropod *Limacina helicina antarctica*. *Conservation Physiology*, 5:1-14.

Iniguez, C., Heinrich, S., Harms, L., and Gordillo, F. J. L. (2017). Increased temperature and $CO_2$ alleviate photoinhibition in *Desmarestia anceps*: from transcriptomics to carbon utilization. *Journal of Experimental Botany*, 68(14):3971–3984.

Karelitz, S. E., Uthicke, S., Foo, S. A., Barker, M. F., Byrne, M., Pecorino, D., and Lamare, M. D. (2016). Ocean acidification has little effect on developmental thermal windows of echinoderms from Antarctica to the tropics. *Global Change Biology*, 23(2):657– 672.

Kawaguchi, S., Ishida, A., King, R., Raymond, B., Waller, N., Constable, A., Nicol, S., Wakita, M., and Ishimatsu, A. (2013). Risk maps for Antarctic krill under projected Southern Ocean acidification. *Nature Climate Change*, 3(9):843–847.

Kawaguchi, S., Kurihara, H., King, R., Hale, L., Berli, T., Robinson, J. P., Ishida, A., Wakita, M., Virtue, P., Nicol, S., and Ishimatsu, A. (2010). Will krill fare well under Southern Ocean acidification? *Biology Letters*, 7(2):288–291.

Koch., F., Beszteri, S., Harms, L., and Trimborn, S. (2019). The impacts of iron limitation and ocean acidification on the cellular stoichiometry, photophysiology, and transcriptome of *Phaeocystis antarctica*. *Limnology and Oceanography*, 64: 357-375.

Maas, E. W., Law, C. S., Hall, J. A., Pickmere, S., Currie, K. I., Chang, F. H., Voyles, K. M., and Caird, D. (2013). Effect of ocean acidification on bacterial abundance, activity and diversity in the Ross Sea, Antarctica. *Aquatic Microbial Ecology*, 70(1):1–15.

McClintock, J. B., Angus, R. A., Mcdonald, M. R., Amsler, C. D., Catledge, S. A., and Vohra, Y. K. (2009). Rapid dissolution of shells of weakly calcified Antarctic benthic macroorganisms indicates high vulnerability to ocean acidification. *Antarctic Science*, 21(5):449–456.

Morley, S. A., Suckling, C. C., Clark, M. S., Cross, E. L., and Peck, L. S. (2016). Long-term effects of altered pH and temperature on the feeding energetics of the Antarctic sea urchin, *Sterechinus neumayeri*. *Biodiversity*, 17(1-2):34–45.

Qu, C.-F., Liu, F.-M., Zheng, Z., Wang, Y.-B., Li, X.-G., Yuan, H.-M., Li, N., An, M.-L., Wang, X.-X., He, Y.-Y., Li, L.-L., and Miao, J.-L. (2017). Effects of ocean acidification on the physiological performance and carbon production of the Antarctic sea ice diatom *Nitzschia* sp. ICE-H. *Marine Pollution Bulletin*, 120(1-2):184–191.

Saba, G. K., Schofield, O., Torres, J. J., Ombres, E. H., and Steinberg, D. K. (2012). Increased feeding and nutrient excretion of adult Antarctic krill, *Euphausia superba*, exposed to enhanced carbon dioxide ($CO_2$). *PLoS ONE*, 7(12).

Schoenrock, K. M., Schram, J. B., Amsler, C. D., McClintock, J. B., Angus, R. A., and Vohra, Y. K. (2016). Climate change confers a potential advantage to fleshy Antarctic crustose macroalgae over calcified species. *Journal of Experimental Marine Biology and Ecology*, 474:58–66.

Schram, J. B., Schoenrock, K. M., McClintock, J. B., Amsler, C. D., and Angus, R. A. (2016a). Testing Antarctic resilience: the effects of elevated seawater temperature and decreased pH on two gastropod species. *ICES Journal of Marine Science*, 73(3): 739-752.

Schram, J. B., Schoenrock, K. M., McClintock, J. B., Amsler, C. D., and Angus, R. A. (2014). Multiple stressor effects of near-future elevated seawater temperature and decreased pH on righting and escape behaviors of two common Antarctic gastropods. *Journal of Experimental Marine Biology and Ecology*, 457:90–96.

Schram, J. B., Schoenrock, K. M., McClintock, J. B., Amsler, C. D., and Angus, R. A. (2016b). Seawater acidification more than warming presents a challenge for two Antarctic macroalgal-associated amphipods. *Marine Ecology Progress Series*, 554:81–97.

Schram, J. B., Schoenrock, K. M., McClintock, J. B., Amsler, C. D., and Angus, R. A. (2017). Ocean warming and acidification alter Antarctic macroalgal biochemical composition but not amphipod grazer feeding preferences. *Marine Ecology Progress Series*, 581:45–56.

Sewell, M.A., Millar, R.B., Yu, P.C., Kapsenberg, L., and Hofmann, G.E. (2014). Ocean acidification and fertilization in the Antarctic sea urchin *Sterechinus neumayeri*: the importance of polyspermy. *Environmental Science & Technology*, 48: 713-722.

Spackeen, J. L., Sipler, R. E., Bertrand, E. M., Xu, K., McQuaid, J. B., Walworth, N. G., Hutchins, D. A., Allen, A. E., and Bronk, D. A. (2018). Impact of temperature, $CO_2$, and iron on nutrient uptake by a late-season microbial community from the Ross Sea, Antarctica. *Aquatic Microbial Ecology*, 82: 145-15.

Strobel, A., Graeve, M., Pörtner, H. O., and Mark, F. C. (2013a). Mitochondrial acclimation capacities to ocean warming and acidification are limited in the Antarctic Nototheniid Fish, *Notothenia rossii* and *Lepidonotothen squamifrons*. *PLoS ONE*, 8(7).

Strobel, A., Leo, E., Pörtner, H. O., and Mark, F. C. (2013b). Elevated temperature and $pCO_2$ shift metabolic pathways in differentially oxidative tissues of *Notothenia rossii*. *Comparative Biochemistry and Physiology, Part B*, 166:48-57.

Strobel, A., Bennecke, S., Leo, E., Mintenbeck, K., Pörtner, H. O., and Mark, F. C. (2012). Metabolic shifts in the Antarctic fish *Notothenia rossii* in response to rising temperature and $pCO_2$. *Frontiers in Zoology*, 9:28.

Thomson, P. G., Davidson, A. T., and Maher, L. (2016). Increasing $CO_2$ changes community composition of pico- and nano-sized protists and prokaryotes at a coastal Antarctic site. *Marine Ecology Progress Series*, 554:51–69.

Torstensson, A., Hedblom, M., Andersson, J., Andersson, M. X., and Wulff, A. (2013). Synergism between elevated $pCO_2$ and temperature on the Antarctic sea ice diatom *Nitzschia lecointei*. *Biogeosciences*, 10:6391–6401.

Tortell, P. D., Payne, C., Gueguen, C., Strzepek, R. F., and Boyd, P. W. (2008a). Inorganic carbon uptake by Southern Ocean phytoplankton. *Limnology and Oceanography*, 53(4):1266–1278.

Tortell, P. D., Payne, C. D., Li, Y., Trimborn, S., Rost, B., Smith, W. O., Riesselman, C., Dunbar, R. B., Sedwick, P., and DiTullio, G. R. (2008b). $CO_2$ sensitivity of Southern Ocean phytoplankton. *Geophysical Research Letters*, 35(4):1–5.

Tortell, P. D., Trimborn, S., Li, Y., Rost, B., and Payne, C. D. (2010). Inorganic carbon utilization by Ross Sea phytoplankton across natural and experimental $CO_2$ gradients. *Journal of Phycology*, 46(3):433– 443.

Trimborn, S., Brenneis, T., Sweet, E., and Rost, B. (2013). Sensitivity of Antarctic phytoplankton species to ocean acidification: growth, carbon acquisition, and species interaction. *Limnology and Oceanography*, 58(3):997–1007.

Trimborn, S., Thoms, S., Brenneis, T., Heiden, J. P., Beszteri, S., and Bischof, K. (2017). Two Southern Ocean diatoms are more sensitive to ocean acidification and changes in irradiance than the prymnesiophyte *Phaeocystis antarctica*. *Physiologia Plantarum*, 160(2): 155–170.

Westwood, K., Thomson, P., van den Enden, R., Ma- her, L., Wright, S., and Davidson, A. (2018). Ocean acidification impacts primary and bacterial production in Antarctic coastal waters during austral summer. *Journal of Experimental Marine Biology and Ecology*, 498:46–60.

Wynn-Edwards, C., King, R., Davidson, A., Wright, S., Nichols, P. D., Wotherspoon, S., Kawaguchi, S., and Virtue, P. (2014). Species-specific variations in the nutritional quality of Southern Ocean phytoplankton in response to elevated $pCO_2$. *Water*, 6(6):1840–1859.

Xu, K., Fu, F.-X., and Hutchins, D. A. (2014). Comparative responses of two dominant Antarctic phytoplankton taxa to interactions between ocean acidification, warming, irradiance, and iron availability. *Limnology and Oceanography*, 59(6):1919–1931.

Young, J. N., Kranz, S. A., Goldman, J. A. L., Tortell, P. D., and Morel, F. M. M. (2015). Antarctic phytoplankton down-regulate their carbon-concentrating mechanisms under high $CO_2$ with no change in growth rates. *Marine Ecology Progress Series*, 532: 13–28.

Yu, P. C., Sewell, M. A., Matson. P. G., Rivest, E. B., Kapsenberg, L., and Hofmann, G. E.

(2013). Growth attenuation with development schedule progression in embryos and early larvae of *Sterechinus neumayeri* raised under elevated $CO_2$. *PLoS One*, 8(1):e52448.

Zhu, Z., Qu, P., Gale, J., Fu, F., and Hutchins, D. A. (2017). Individual and interactive effects of warming and $CO_2$ on *Pseudo-nitzschia subcurvata* and *Phaeocystis antarctica*, two dominant phytoplankton from the Ross Sea, Antarctica. *Biogeosciences*,14:5281-5295.

**Papers not included in the meta-analysis and the reasoning behind omission**

Boelen, P., van de Poll, W. H., van der Strate, H. J., Neven, I. A., Beardall, J., and Buma, A. G. J. (2011). Neither elevated nor reduced $CO_2$ affects the photophysiological performance of the marine Antarctic diatom *Chaetoceros brevis*. *Journal of Experimental Marine Biology and Ecology*, 406: 38-45
- Not included as there was no ambient $CO_2$ treatment, on elevated (790 ppmv) or reduced (190 ppmv) $CO_2$ treatments.

Benedetti, M., Lanzoni, I., Nardi, A., d'Errico, G., Di Carlo, M., Fattorini, D., Nigro, M., and Regoli, F. (2016). Oxidative responsiveness to multiple stressors in key Antarctic species, *Adamussium colbecki*: interactions between temperature, acidification and cadmium exposure. *Marine Environmental Research*, 121: 20-30.
- Not included as carbonate chemistry measurements required to convert pH (measured in the paper) to $CO_2$ ($\mu$atm) not provided in the paper and the corresponding author could not be contacted.

Beszteri, S., Thoms, S., Benes, V., Harms, L., and Trimborn, S. (2018). The response of three Southern Ocean phytoplankton species to ocean acidification and light availability: a transcriptomic study. *Protist*, 169: 958-975.
- Not included as this is a transcriptomic study and does not have usable measurements (mean and standard error) to input into meta-analytic statistical analyses.

Castrisios, K., Martin, A., Muller, M. N., Kennedy, F., McMinn, A., and Ryan, K. G. (2018). Response of Antarctic sea-ice algae to an experimental decrease in pH: a preliminary analysis from chlorophyll fluorescence imaging of melting ice. *Polar Research*, 37(1): 1438696.
- Not included as this was a sea-ice study with a high ambient $CO_2$ treatment (1651 $\mu$atm).

Cummings, V., Hwitt, J., Van Rooyen, A., Currie, K., Beard, S., Thrush, S., Norkko. J., Barr, N., Heath, P., Halliday, N. J., Sedcole, R., Gomez, A., McGraw, C., and Metcalf, V. (2011). Ocean acidification at high latitudes: potential effects of functioning of the Antarctic bivalve *Laternula elliptica*. *PLoS ONE*, 6(1):e16069.
- Not included as this is a genomic study and does not have usable measurements (mean and standard error) to input into meta-analytic statistical analyses.

Endo, H., Hattori, H., Mishima, T., Hashida, G., Sasaki, H., Nishioka, J., and Suzuki, K. (2017). Phytoplankton community responses to iron and $CO_2$ enrichment in different biogeochemical regions of the Southern Ocean. *Polar Biology*, 40: 2143-2159.
- Not included as there is no ambient iron with manipulated $CO_2$ treatment; there are only control, iron added and iron + $CO_2$ added treatments.

Enzor, L. A., and Place, S. P. (2014). Is warmer better? Decreased oxidative damage in notothenioid fish after long-term acclimation to multiple stressors. *The Journal of Experimental Biology*, 217:3301-3310.
- Not included as no biological measurements presented in the article were comparable to measurements in other fish studies.

Ericson, J. A., Hellessey, N., Kawaguchi, S., Nicol, S., Hoem, N., and Virtue, P. (2018). Adult Antarctic krill proves resilient in a simulated high $CO_2$ ocean. *Nature Communications*, 1(1):190.
- Not included as this is a long-term study (46 weeks) investigating the acclimative capacity of Antarctic krill to ocean acidification.

Hoppe, C. J. M., Hassler, C. S., Payne, C. D., Tortell, P. D., Rost, B., and Trimborn, S. (2013.) Iron limitation modulates ocean acidification effect on Southern Ocean phytoplankton communities. *PLoS ONE*, 8(11):e79890.
- Not included as this was a multistressor experiment investigating the effect of ocean acidification and iron availability. There was no ambient iron treatment only iron enriched and iron deplete where hydroxamate siderophore desferrioxamine was added to bind and reduce the bioavailability of iron.

Huth, T., and Place, S. (2016). RNA-seq reveals a diminished acclimation response to the combined effects of ocean acidification and elevated temperature in *Pagothenia borchgrevinki*. *Marine Genomics*, 28:87-97.
- Not included as this is a transcriptomic study and does not have usable measurements (mean and standard error) to input into meta-analytic statistical analyses.

Huth, T., and Place, S. (2016). Transcriptome wide analysis reveal a sustained cellular stress response in the gill tissue of *Trematomus bernacchii* after acclimation to multiple stressors. *BMC Genomics*, 17:127.
- Not included as this is a transcriptomic study and does not have usable measurements (mean and standard error) to input into meta-analytic statistical analyses.

Johnson, K. M., and Hofmann, G. E. (2017). Transciptomic response of the Antarctic pteropod *Limacina helicina antarctica* to ocean acidification. *BMC Genomics*, 18: 212.
- Not included as this is a transcriptomic study and does not have usable measurements (mean and standard error) to input into meta-analytic statistical analyses.

Kapsenberg, L., and Hofmann, G. E. (2014). Signals of resilience to ocean change: high thermal tolerance of early stage Antarctic sea urchins (*Sterechinus neumayeri*) reared under present-day and future $pCO_2$ and temperature. *Polar Biology*, 37: 967-950.
- Not included as there was no ambient temperature treatment. The effect of increased $CO_2$ over temperature gradients was investigated in this paper.

McMinn, A., Muller, M. N., Martin, A., Ugalde, S. C., Lee, S., Castrisios, K., and Ryan, K. G. (2017). Effect of $CO_2$ concentration on a late summer surface sea ice community. *Marine Biology*, 164: 87.
- Not included as this is a sea-ice study with a high ambient $CO_2$ treatment (1288 $\mu$atm).

McMinn, A., Muller, M. N., Martin, A., and Ryan, K. G. (2014). The response of Antarctic sea ice algae to changes in pH and $CO_2$. *PLoS ONE*, 9(1): e86984.
- Not included as this is a sea-ice study with a high ambient $CO_2$ treatment (1190 $\mu$atm).

Mutungi, G., and Johnston, I. A. (1988). Influence of pH and temperature on force development and shortening velocity in skinned muscle fibres from fish. *Fish Physiology and Biochemistry*, 5(4): 257-262.
- Not included as carbonate chemistry measurements required to convert pH (measured in the paper) to $CO_2$ ($\mu$atm) not provided in the paper and the corresponding author could not be contacted.

Schram, J. B., Amsler, M. O., Amsler, C. D., Schoenrock, K. M., McClintock, J. B., and Angus, R. A. (2016a). Antarctic crustacean grazer assemblages exhibit resistance following exposure to decreased pH. *Marine Biology*, 163(5).
- Not included as looking at crustacean grazer assemblage change and does not have usable measurements (mean and standard error) to input into meta-analytic statistical analyses.

Torstensson, M., Hedblom, M., Björk, M. M., Chierici, M., and Wulff, A. (2015). Long-term acclimation to elevated $pCO_2$ alters carbon metabolism and reduces growth in the Antarctic diatom *Nitzschia lecointei*. *Proceedings of the Royal Scoiety B: Biological Sciences*, 282: 20151513.
- Not included as this is a long-term study (147 days) investigating the acclimative capacity of Antarctic phytoplankton to ocean acidification.

Trimborn, S., Thoms, S., Petrou, K., Kranz, S. A., and Rost, B. (2014). Photophysiological responses of Southern Ocean phytoplankton to changes in $CO_2$ concentrations: short-term versus acclimation effects. *Journal of Experimental Marine Biology and Ecology*, 451: 44-54.
- Not included as this study measured response curves of low vs high $CO_2$ acclimated phytoplankton cultures and was therefore uncomparable to other studies included in the meta-analysis.

Yang, G., King, R. A., and Kawaguchi, S. (2018). Behavioural responses of Antarctic krill (*Euphausia superba*) to $CO_2$-induced ocean acidification: would krill really notice? *Polar Biology*, 41: 727-732.
- Not included as there are no usable measurement (mean and standard error) to input into meta-analytic statistical analyses.

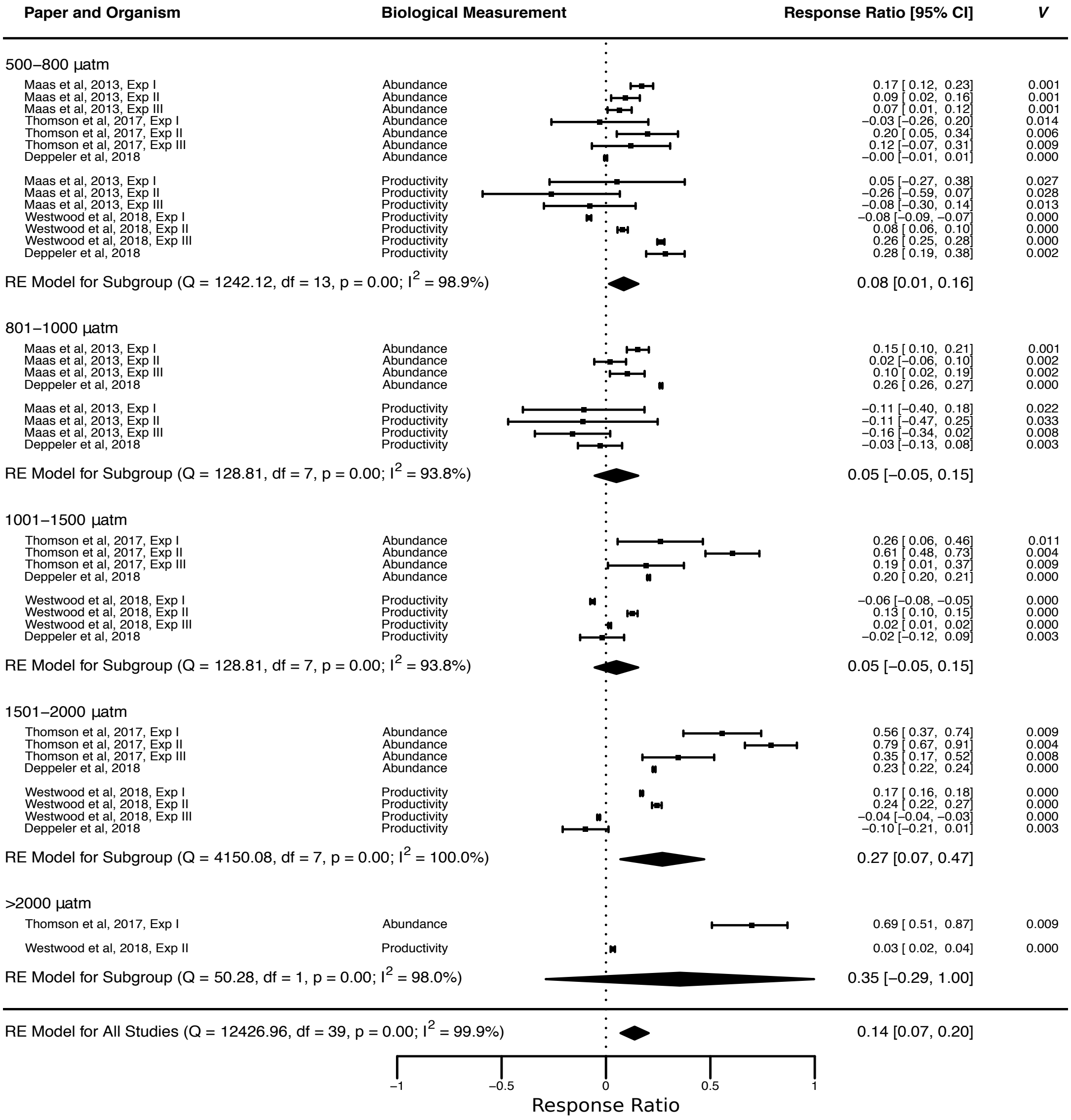


Figure S1: Forest plots of all bacterial response ratios and variance included in the meta-analysis. The data is separated by $CO_2$ treatment at which the response was measured and information is provided on the study paper, experiment and biological measurement from which the response ratio is calculated. At the end of each $CO_2$ bracket summary statistics from weighted, random effects are provided including the Q statistic, degrees of freedom, p-value, $I^2$, mean response ratio and 95% confidence interval.

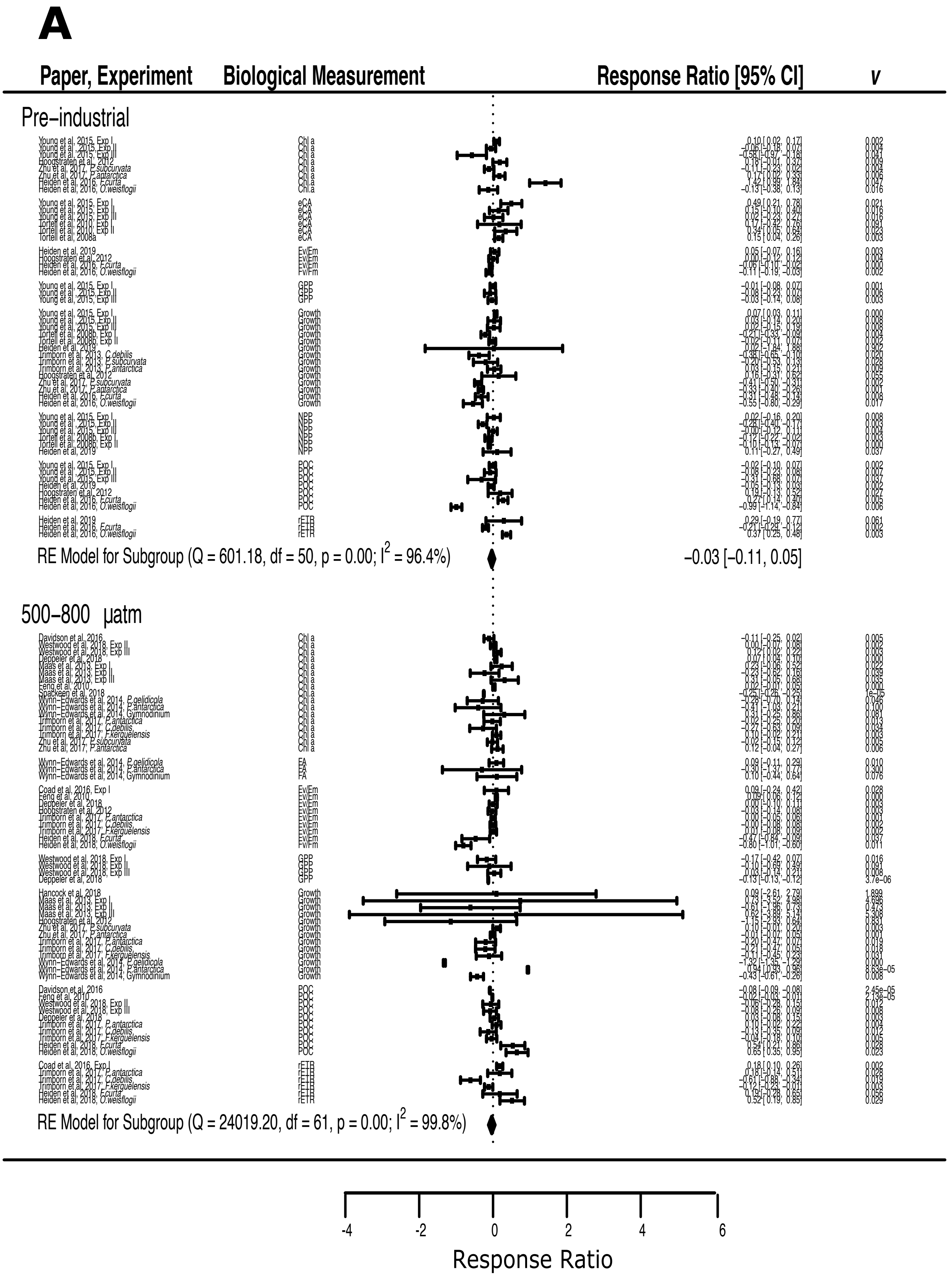


Figure S2: Forest plot of all phytoplankton response ratios, 95% confidence intervals and variance (v) included in the meta-analysis.

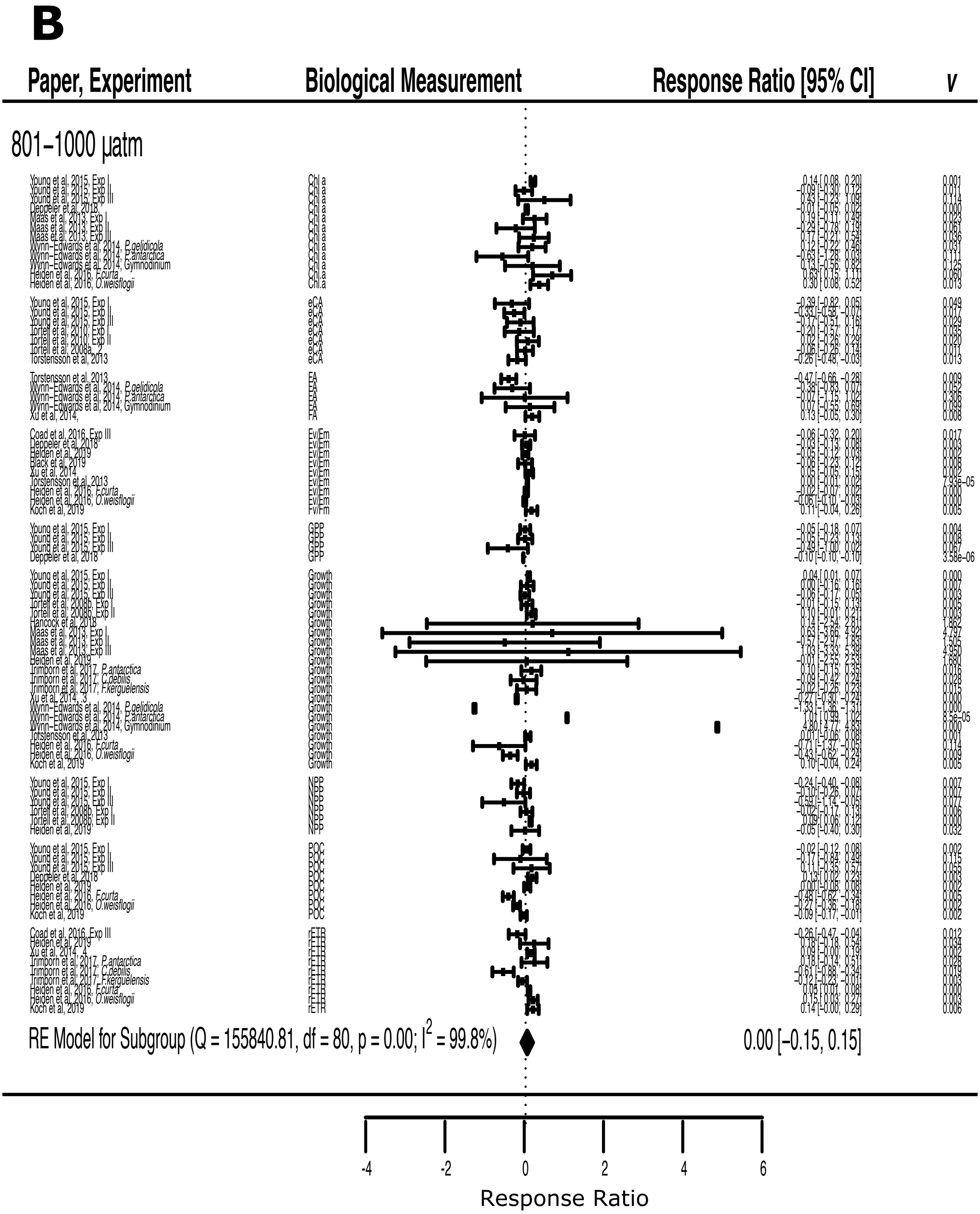


Figure S2: Forest plot of all phytoplankton response ratios, 95% confidence intervals and variance (v) included in the meta-analysis.

**c**

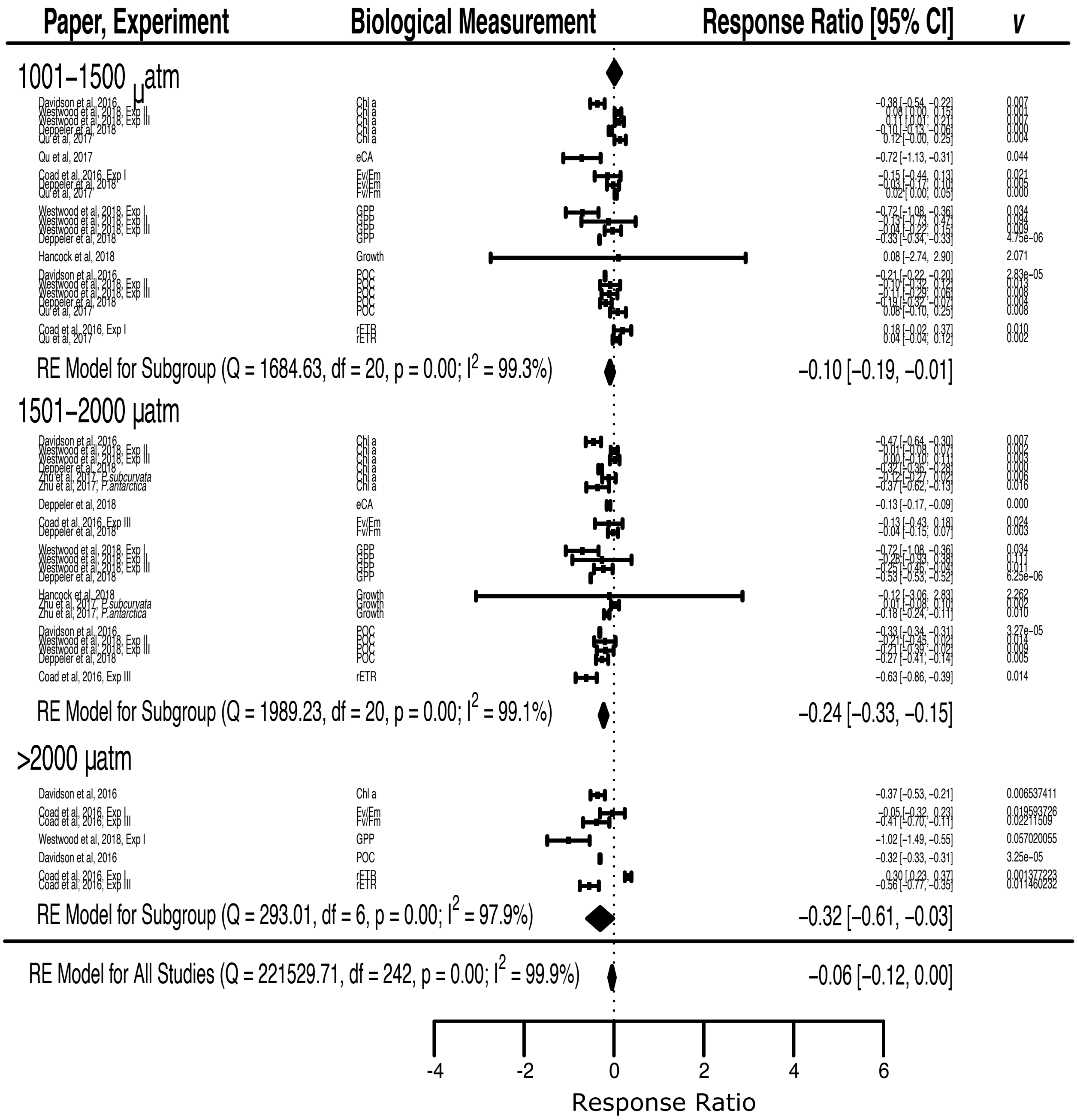


Figure S2: Forest plot of all phytoplankton response ratios, 95% confidence intervals and variance (v) included in the meta-analysis (A: pre-industrial and 500-800 $\mu$atm, B: 801-1000 $\mu$atm, C: 1001-1500, 1501-2000 and >2000 $\mu$atm). The data is separated by $CO_2$ level at which the response was measured and information is provided on the study paper and biological response from which the response ratio is calculated. At the end of each $CO_2$ level summary statistics from weighted random effects models are provided including the Q statistic, degrees of freedom, p-value, $I^2$, mean response ratio and 95% confidence interval. Summary statistics are also provided at the bottom of the figure for all phytoplankton response ratios with all $CO_2$ levels together.

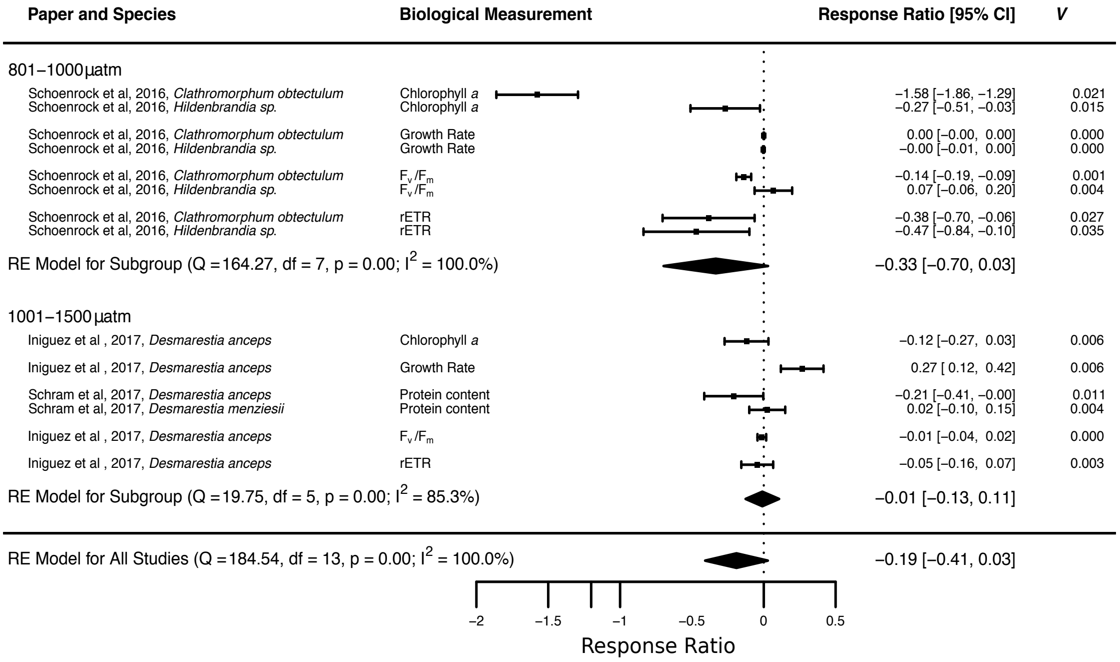


Figure S3: Forest plots of all macroalgal response ratios and variance included in the meta-analysis. The data is separated by $CO_2$ treatment at which the response was measured and information is provided on the study paper, species and biological measurement from which the response ratio is calculated. At the end of each $CO_2$ bracket summary statistics from weighted, random effects are provided including the Q statistic, degrees of freedom, p-value, $I^2$, mean response ratio and 95% confidence interval.

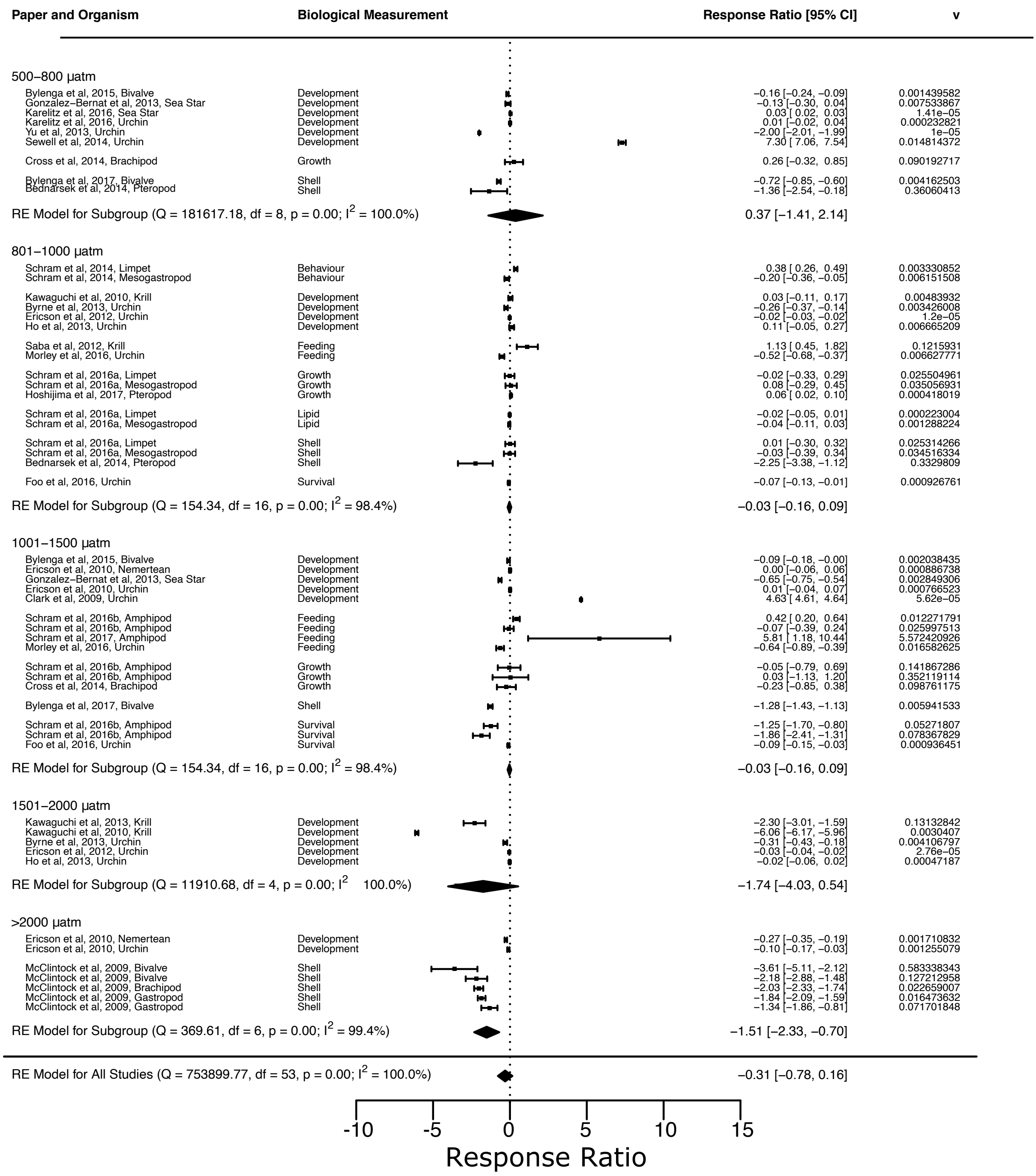


Figure S4: Forest plots of all invertebrate response ratios and variance included in the meta-analysis. The data is separated by $CO_2$ treatment at which the response was measured and information is provided on the study paper, organism and biological measurement from which the response ratio is calculated. At the end of each $CO_2$ bracket summary statistics from weighted, random effects are provided including the Q statistic, degrees of freedom, p-value, $I^2$, mean response ratio and 95% confidence interval.

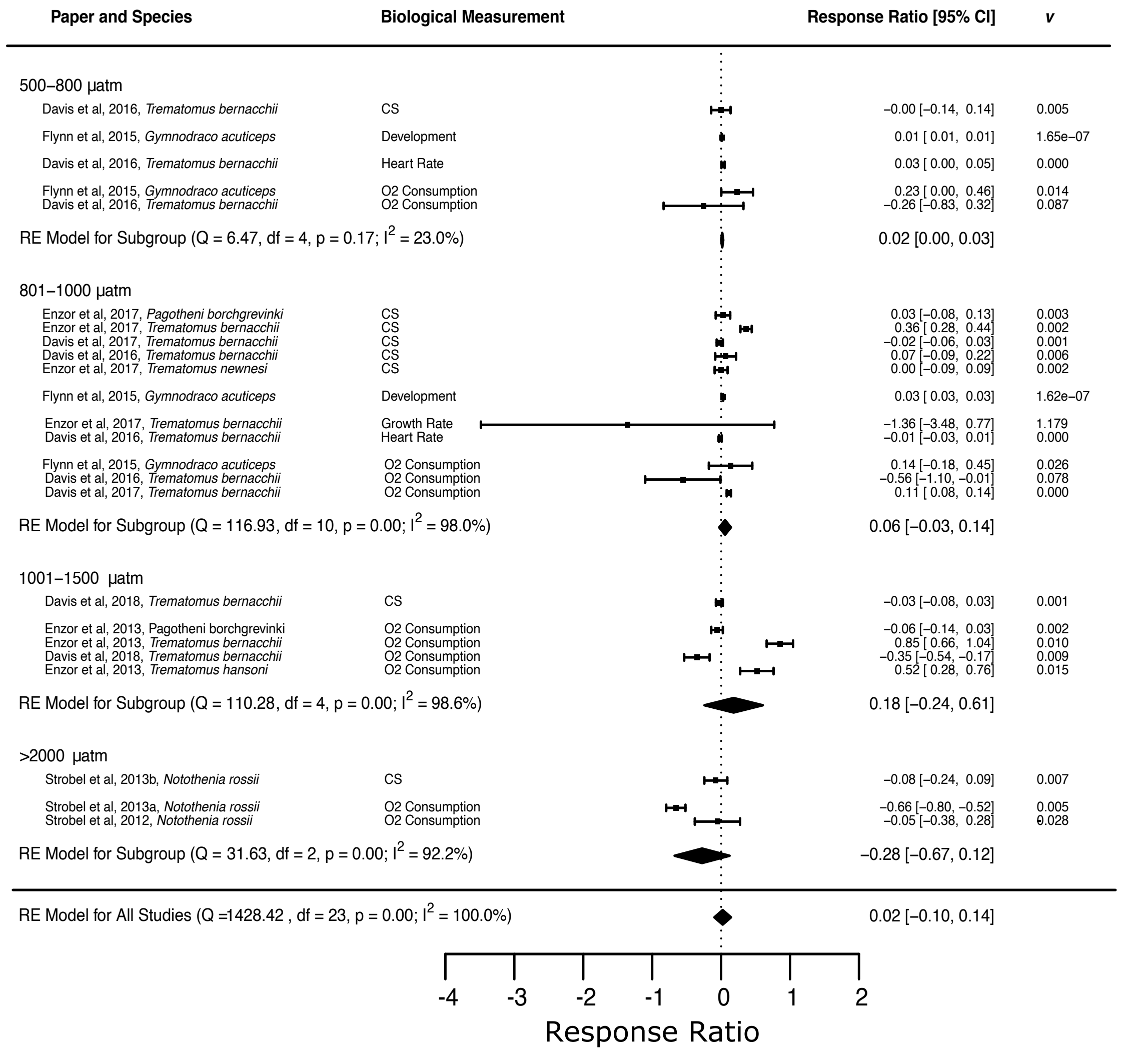


Figure S5: Forest plots of all fish response ratios and variance included in the meta-analysis. The data is separated by $CO_2$ treatment at which the response was measured and information is provided on the study paper, species and biological measurement from which the response ratio is calculated. At the end of each $CO_2$ bracket summary statistics from weighted, random effects are provided including the Q statistic, degrees of freedom, p-value, $I^2$, mean response ratio and 95% confidence interval.

Table S2: Q test and random effects model results for bacteria.

| | Q-Test Results | | | Model Results |
|---|---|---|---|---|
| | df | Q | p | |
| *per $CO_2$ Level* | | | | |
| All levels | 39 | 12427.0 | <0.0001 | Positive |
| 500-800 $\mu$atm | 13 | 1242.1 | <0.0001 | Positive |
| 801-1000 $\mu$atm | 7 | 128.8 | <0.0001 | Positive |
| 1001-1500 $\mu$atm | 7 | 2696.9 | <0.0001 | Positive |
| 1501-2000 $\mu$atm | 7 | 4150.1 | <0.0001 | Positive |
| >2000 $\mu$atm | 7 | 50.3 | <0.0001 | Positive |
| *per Biological Measurement* | | | | |
| Abundance | 19 | 4505.4 | <0.0001 | Positive |
| Productivity | 19 | 4201.6 | <0.0001 | No effect |
| *per $CO_2$ Level and Biological Measurement* | | | | |
| Abundance | | | | |
| 500-800 $\mu$atm | 6 | 58.6 | <0.0001 | Positive |
| 801-1000 $\mu$atm | 3 | 69.5 | <0.0001 | Positive |
| 1001-1500 $\mu$atm | 3 | 37.2 | <0.0001 | Positive |
| 1501-2000 $\mu$atm | 3 | 90.6 | <0.0001 | Positive |
| >2000 $\mu$atm | 1 study | N/A | | Positive |
| Productivity | | | | |
| 500-800 $\mu$atm | 6 | 1163.5 | <0.0001 | No effect |
| 801-1000 $\mu$atm | 3 | 1.7 | **0.647** | Negative |
| 1001-1500 $\mu$atm | 3 | 275.0 | <0.0001 | No effect |
| 1501-2000 $\mu$atm | 3 | 2334.5 | <0.0001 | Positive |
| >2000 $\mu$atm | 1 study | N/A | | Positive |

Table S3: Q test and random effects model results for phytoplankton. $CO_2$ levels are in $\mu$atm, Pos = positive effect, Neg = negative effect, and NE = no effect.

| | Q-Test Results | | | Model Results | | Q-Test Results | | | Model Results | | Q-Test Results | | | Model Results |
|---|---|---|---|---|---|---|---|---|---|---|---|---|---|---|
| | df | Q | p | | | df | Q | p | | | df | Q | p | |
| *per $CO_2$ Level* | | | | | *per $CO_2$ Level (Single Species Studies Only)* | | | | | *per $CO_2$ Level (Community Level Studies Only)* | | | | |
| All levels | 242 | 1529.7 | <0.0001 | Neg | All levels | 102 | 161284.2 | <0.0001 | NE | All levels | 139 | 1201.1 | <0.0001 | Neg |
| Pre-industrial | 50 | 601.2 | <0.0001 | NE | Pre-industrial | 20 | 531.1 | <0.0001 | NE | Pre-industrial | 29 | 38.2 | <0.0001 | NE |
| 500-800 | 60 | 21850.6 | <0.0001 | NE | 500-800 | 35 | 19367.7 | <0.0001 | NE | 500-800 | 24 | 994.9 | <0.0001 | NE |
| 801-1000 | 80 | 155840.8 | <0.0001 | NE | 801-1000 | 36 | 135163.8 | <0.0001 | NE | 801-1000 | 43 | 374.1 | <0.0001 | Neg |
| 1001-1500 | 20 | 1684.6 | <0.0001 | Neg | 1001-1500 | 4 | 15.3 | 0.0042 | NE | 1001-1500 | 15 | 840.8 | <0.0001 | Neg |
| 1501-2000 | 20 | 1989.2 | <0.0001 | Neg | 1501-2000 | 3 | 15.5 | 0.0014 | Neg | 1501-2000 | 16 | 1749.1 | <0.0001 | Neg |
| >2000 | 6 | 293.0 | <0.0001 | Neg | >2000 | 0 studies | | N/A | | >2000 | 6 | 293.0 | <0.0001 | Neg |
| *per Biological Measurement* | | | | | *per Biological Measurement(Single Species Studies Only)* | | | | | *per Biological Measurement(Community Level Studies Only)* | | | | |
| Chlorophyll *a* | 48 | 1610.2 | <0.0001 | NE | Chlorophyll *a* | 20 | 94.8 | <0.0001 | NE | Chlorophyll *a* | 27 | 1385.9 | <0.0001 | NE |
| Growth | 51 | 152844.1 | <0.0001 | NE | Growth | 29 | 14727.9 | <0.0001 | NE | Growth | 21 | 28.1 | **0.1380** | NE |
| GPP | 19 | 23994.4 | <0.0001 | Neg | GPP | 0 studies | | N/A | | GPP | 19 | 23994.4 | <0.0001 | Neg |
| NPP | 11 | 112.8 | <0.0001 | Neg | NPP | 0 studies | | N/A | | NPP | 11 | 112.9 | <0.0001 | Neg |
| $F_v/F_m$ | 28 | 148.7 | <0.0001 | Neg | $F_v/F_m$ | 14 | 97.1 | <0.0001 | NE | $F_v/F_m$ | 13 | 35.1 | 0.0008 | NE |
| rETR | 22 | 277.4 | <0.0001 | NE | rETR | 14 | 143.9 | <0.0001 | NE | rETR | 7 | 117.1 | <0.0001 | NE |
| POC | 34 | 3290.4 | <0.0001 | Neg | POC | 11 | 267.5 | <0.0001 | NE | POC | 22 | 3019.9 | <0.0001 | Neg |
| Fatty Acids | 7 | 26.7 | 0.0004 | NE | Fatty Acidst | 7 | 26.7 | 0.004 | NE | Fatty Acids | 0 studies | | N/A | |
| eCA | 14 | 70.2 | <0.0001 | NE | eCA | 1 | 3.8 | **0.0517** | Neg | eCA | 12 | 58.8 | <0.0001 | Neg |

Table S.3 continued.

| | Q-Test Results | | | Model Results | | Q-Test Results | | | Model Results | | Q-Test Results | | | Model Results |
|---|---|---|---|---|---|---|---|---|---|---|---|---|---|---|
| | df | Q | p | | | df | Q | p | | | df | Q | p | |
| *per $CO_2$ Level and Biological Measurement* | | | | | *per $CO_2$ Level and Biological Measurement (Single Species Studies Only)* | | | | | *per $CO_2$ Level and Biological Measurement (Community Level Studies Only)* | | | | |
| Chlorophyll *a* | | | | | Chlorophyll *a* | | | | | Chlorophyll *a* | | | | |
| Pre-industrial | 7 | 65.2 | <0.0001 | NE | Pre-industrial | 4 | 50.9 | <0.0001 | NE | Pre-industrial | 2 | 13.7 | 0.0011 | NE |
| 500-800 | 15 | 29.8 | 0.0125 | Pos | 500-800 | 7 | 10.7 | **0.1511** | NE | 500-800 | 7 | 19.0 | 0.0081 | Pos |
| 801-1000 | 11 | 42.2 | <0.0001 | NE | 801-1000 | 4 | 10.1 | **0.0382** | NE | 801-1000 | 6 | 26.0 | 0.0002 | Pos |
| 1001-1500 | 4 | 51.0 | <0.0001 | NE | 1001-1500 | 1 study | | N/A | Pos | 1001-1500 | 3 | 43.1 | <0.0001 | NE |
| 1501-2000 | 5 | 81.6 | <0.0001 | Neg | 1501-2000 | 1 | 2.9 | **0.0909** | Neg | 1501-2000 | 3 | 78.2 | <0.0001 | Neg |
| >2000 | 1 study | | N/A | Neg | >2000 | 0 studies | | N/A | | >2000 | 1 study | | N/A | Neg |
| Growth | | | | | Growth | | | | | Growth | | | | |
| Pre-industrial | 13 | 90.5 | <0.0001 | Neg | Pre-industrial | 7 | 25.2 | 0.0007 | Neg | Pre-industrial | 5 | 21.6 | 0.0006 | NE |
| 500-800 | 12 | 19061.2 | <0.0001 | Neg | 500-800 | 8 | 19059.7 | <0.0001 | NE | 500-800 | 3 | 0.7 | **0.8792** | NE |
| 801-1000 | 20 | 123630.7 | <0.0001 | NE | 801-1000 | 10 | 1189006.2 | <0.0001 | NE | 801-1000 | 9 | 5.4 | **0.7966** | NE |
| 1001-1500 | 1 study | | N/A | NE | 1001-1500 | 0 studies | | N/A | | 1001-1500 | 1 study | | N/A | NE |
| 1501-2000 | 2 | 11.5 | 0.0032 | NE | 1501-2000 | 1 | 11.5 | 0.0007 | NE | 1501-2000 | 1 study | | N/A | NE |
| >2000 | 0 studies | | N/A | | >2000 | 0 studies | | N/A | | >2000 | 0 studies | | N/A | |
| GPP | | | | | | | | | | GPP | | | | |
| Pre-industrial | 2 | 0.7 | **0.7016** | NE | | | | | | Pre-industrial | 2 | 0.7 | **0.7016** | NE |
| 500-800 | 3 | 3.4 | **0.3374** | Neg | | | | | | 500-800 | 3 | 3.4 | **0.3374** | Neg |
| 801-1000 | 3 | 3.1 | **0.3797** | Neg | | | | | | 801-1000 | 3 | 3.1 | **0.3797** | Neg |
| 1001-1500 | 3 | 14.7 | 0.0021 | Neg | | | | | | 1001-1500 | 3 | 14.7 | 0.0021 | Neg |
| 1501-2000 | 3 | 8.5 | 0.0361 | Neg | | | | | | 1501-2000 | 3 | 8.5 | 0.0361 | Neg |
| >2000 | 1 study | | N/A | Neg | | | | | | >2000 | 1 study | | N/A | Neg |

Table S.3 continued.

| *per $CO_2$ Level and Biological Measurement* | Q-Test Results df | Q | p | Model Results |
|---|---|---|---|---|
| NPP | | | | |
| Pre-industrial | 5 | 15.9 | **0.0072** | Neg |
| 500-800 | 5 | 26.6 | <0.0001 | Neg |
| 801-1000 | 0 studies | | N/A | |
| 1001-1500 | 0 studies | | N/A | |
| 1501-2000 | 0 studies | | N/A | |
| >2000 | 0 studies | | N/A | |
| $F_v/F_m$ | | | | |
| Pre-industrial | 3 | 5.8 | **0.1231** | NE |
| 500-800 | 8 | 86.4 | <0.0001 | NE |
| 801-1000 | 8 | 16.3 | 0.0383 | NE |
| 1001-1500 | 2 | 2.2 | **0.336** | NE |
| 1501-2000 | 1 | 0.3 | **0.5769** | NE |
| >2000 | 1 | 3.1 | **0.0807** | Neg |
| rETR | | | | |
| Pre-industrial | 0 studies | | N/A | |
| 500-800 | 5 | 49.8 | <0.0001 | NE |
| 801-1000 | 8 | 45.7 | <0.0001 | NE |
| 1001-1500 | 1 | 1.6 | **0.2069** | NE |
| 1501-2000 | 1 study | | N/A | Neg |
| >2000 | 1 | 57.5 | <0.0001 | NE |

| *per $CO_2$ Level and Biological Measurement (Single Species Studies Only)* | Q-Test Results df | Q | p | Model Results |
|---|---|---|---|---|
| $F_v/F_m$ | | | | |
| Pre-industrial | 2 | 2.4 | **0.2998** | Neg |
| 500-800 | 5 | 60.8 | <0.0001 | NE |
| 801-1000 | 4 | 14.7 | 0.0053 | NE |
| 1001-1500 | 1 study | | N/A | Pos |
| 1501-2000 | 0 studies | | N/A | |
| >2000 | 0 studies | | N/A | |
| rETR | | | | |
| Pre-industrial | 0 studies | | N/A | |
| 500-800 | 4 | 31.6 | <0.0001 | NE |
| 801-1000 | 6 | 37.6 | <0.0001 | NE |
| 1001-1500 | 1 study | | N/A | NE |
| 1501-2000 | 0 studies | | N/A | |
| >2000 | 0 studies | | N/A | |

| *per $CO_2$ Level and Biological Measurement (Community Level Studies Only)* | Q-Test Results df | Q | p | Model Results |
|---|---|---|---|---|
| NPP | | | | |
| Pre-industrial | 5 | 15.9 | **0.0072** | Neg |
| 500-800 | 5 | 26.6 | <0.0001 | Neg |
| 801-1000 | 0 studies | | N/A | |
| 1001-1500 | 0 studies | | N/A | |
| 1501-2000 | 0 studies | | N/A | |
| >2000 | 0 studies | | N/A | |
| $F_v/F_m$ | | | | |
| Pre-industrial | 1 study | | N/A | NE |
| 500-800 | 2 | 2.2 | **0.3307** | Pos |
| 801-1000 | 3 | 0.1 | **0.9872** | NE |
| 1001-1500 | 1 | 0.6 | **0.4573** | NE |
| 1501-2000 | 1 | 0.3 | **0.5769** | NE |
| >2000 | 1 | 3.1 | **0.0807** | Neg |
| rETR | | | | |
| Pre-industrial | 0 studies | | N/A | |
| 500-800 | 1 study | | N/A | Pos |
| 801-1000 | 1 | 4.1 | 0.0418 | Neg |
| 1001-1500 | 1 study | | N/A | Pos |
| 1501-2000 | 1 study | | N/A | Neg |
| >2000 | 1 | 57.4 | <0.0001 | NE |

Table S.3 continued.

| *per $CO_2$ Level and Biological Measurement* | Q-Test Results df | Q | p | Model Results |
|---|---|---|---|---|
| POC | | | | |
| Pre-industrial | 6 | 179.6 | <0.0001 | NE |
| 500-800 | 9 | 144.7 | <0.0001 | NE |
| 801-1000 | 7 | 68.8 | <0.0001 | NE |
| 1001-1500 | 4 | 12.6 | 0.0132 | Neg |
| 1501-2000 | 3 | 3.0 | **0.3976** | Neg |
| >2000 | 1 study | | N/A | Neg |
| | | | | |
| eCA | | | | |
| Pre-industrial | 5 | 7.8 | **0.1703** | Pos |
| 500-800 | 0 studies | | N/A | |
| 801-1000 | 6 | 5.9 | **0.4318** | Neg |
| 1001-1500 | 1 study | | N/A | Neg |
| 1501-2000 | 1 study | | N/A | Neg |
| >2000 | 0 studies | | N/A | |
| | | | | |
| Fatty Acids | | | | |
| Pre-industrial | 0 studies | | N/A | |
| 500-800 | 2 | 0.5 | **0.7806** | NE |
| 801-1000 | 4 | 21.9 | 0.0002 | Neg |
| 1001-1500 | 0 studies | | N/A | |
| 1501-2000 | 0 studies | | N/A | |
| >2000 | 0 studies | | N/A | |

| *per $CO_2$ Level and Biological Measurement (Single Species Studies Only)* | Q-Test Results df | Q | p | Model Results |
|---|---|---|---|---|
| POC | | | | |
| Pre-industrial | 2 | 162.9 | <0.0001 | NE |
| 500-800 | 4 | 27.9 | <0.0001 | NE |
| 801-1000 | 0 studies | | N/A | |
| 1001-1500 | 1 study | | N/A | NE |
| 1501-2000 | 0 studies | | N/A | |
| >2000 | 0 studies | | N/A | |
| | | | | |
| eCA | | | | |
| Pre-industrial | 0 studies | | N/A | |
| 500-800 | 0 studies | | N/A | |
| 801-1000 | 1 study | | N/A | Neg |
| 1001-1500 | 1 study | | N/A | Neg |
| 1501-2000 | 0 studies | | N/A | |
| >2000 | 0 studies | | N/A | |
| | | | | |
| Fatty Acids | | | | |
| Pre-industrial | 0 studies | | N/A | |
| 500-800 | 2 | 0.5 | **0.7806** | NE |
| 801-1000 | 4 | 21.9 | 0.0002 | Neg |
| 1001-1500 | 0 studies | | N/A | |
| 1501-2000 | 0 studies | | N/A | |
| >2000 | 0 studies | | N/A | |

| *per $CO_2$ Level and Biological Measurement (Community Level Studies Only)* | Q-Test Results df | Q | p | Model Results |
|---|---|---|---|---|
| POC | | | | |
| Pre-industrial | 3 | 2.4 | **0.4892** | Neg |
| 500-800 | 4 | 105.3 | <0.0001 | Neg |
| 801-1000 | 4 | 4.9 | **0.3009** | NE |
| 1001-1500 | 3 | 2.2 | **0.5200** | Neg |
| 1501-2000 | 3 | 3.0 | **0.3976** | Neg |
| >2000 | 1 study | | N/A | Neg |
| | | | | |
| eCA | | | | |
| Pre-industrial | 5 | 7.8 | **0.1703** | Pos |
| 500-800 | 0 studies | | N/A | |
| 801-1000 | 5 | 5.3 | **0.3853** | Neg |
| 1001-1500 | 0 studies | | N/A | |
| 1501-2000 | 1 study | | N/A | Neg |
| >2000 | 0 studies | | N/A | |

Table S4: Q test and random effects model results for macroalgae.

| | Q-Test Results | | | Model Results |
|---|---|---|---|---|
| | df | Q | p | |
| *per $CO_2$ Level* | | | | |
| All levels | 13 | 184.5 | <0.0001 | Negative |
| 801-1000 $\mu$atm | 7 | 164.3 | <0.0001 | Negative |
| 1001-1500$mu$atm | 5 | 19.7 | 0.0014 | No effect |
| *per Biological Measurement* | | | | |
| Chlorophyll *a* concentration | 2 | 80.0 | <0.0001 | Negative |
| Growth Rate | 2 | 13.5 | 0.0012 | No effect |
| Protein content | 1 | 3.6 | **0.0578** | No effect |
| rETR | 2 | 7.7 | 0.0208 | Negative |
| $F_v/F_m$ | 2 | 19.6 | <0.0001 | No effect |
| *per $CO_2$ Level and Biological Measurement* | | | | |
| Chlorophyll *a* concentration | | | | |
| 801-1000 $\mu$atm | 1 | 47.3 | <0.0001 | No effect |
| 1001-1500 $\mu$atm | 1 study | N/A | | No effect |
| Growth Rate | | | | |
| 801-1000 $\mu$atm | 1 | 1.0 | **0.3232** | No effect |
| 1001-1500 $\mu$atm | 1 study | N/A | | Positive |
| Protein Content | | | | |
| 801-1000 $\mu$atm | 0 studies | N/A | | |
| 1001-1500 $\mu$atm | 11 | 3.6 | **0.0578** | No effect |
| rETR | | | | |
| 801-1000 $\mu$atm | 1 | 0.1 | **0.7347** | Negative |
| 1001-1500 $\mu$atm | 1 study | N/A | | No effect |
| $F_v/F_m$ | | | | |
| 801-1000 $\mu$atm | 1 | 8.2 | 0.0041 | No effect |
| 1001-1500 $\mu$atm | 1 study | N/A | | No effect |
| *per Species* | | | | |
| Clathromorphum obtectulum | 3 | 151.7 | <0.0001 | Negative |
| (only studied at 801-1000 $\mu$atm) | | | | |
| Desmarestia anceps | 4 | 19.4 | 0.0006 | No effect |
| (only studied at 1001-1500 $\mu$atm) | | | | |
| Desmarestia menziesii | 1 study | N/A | | No effect |
| (only studied at 1001-1500 $\mu$atm) | | | | |
| Hildenbrandia sp. | 3 | 11.9 | 0.0079 | Negative |
| (only studied at 801-1000 $\mu$atm) | | | | |

Table S5: Q test and random effects model results for invertebrates.

| | Q-Test Results | | | Model Results |
|---|---|---|---|---|
| | df | Q | p | |
| *per $CO_2$ Level* | | | | |
| All levels | 55 | 754524.3 | <0.0001 | Negative |
| 500-800 $\mu$atm | 8 | 181617.2 | <0.0001 | No effect |
| 801-1000 $\mu$atm | 18 | 218.3 | <0.0001 | No effect |
| 1001-1500 $\mu$atm | 15 | 509.5 | <0.0001 | Negative |
| 1501-2000 $\mu$atm | 4 | 11910.7 | <0.0001 | Negative |
| >2000 $\mu$atm | 6 | 369.6 | <0.0001 | Negative |
| | | | | |
| *per Biological Measurement* | | | | |
| Development | 21 | 751805.0 | <0.0001 | No effect |
| Behaviour | 1 | 35.4 | <0.0001 | No effect |
| Feeding | 5 | 80.6 | <0.0001 | No effect |
| Growth Rate | 6 | 1.6 | **0.9496** | Positive |
| Lipid and Protein Content | 1 | 0.4 | **0.5191** | No effect |
| Shell State | 10 | 216.8 | <0.0001 | Negative |
| Survival | 3 | 65.8 | <0.0001 | Negative |
| | | | | |
| *per $CO_2$ Level and Biological Measurement* | | | | |
| Development | | | | |
| 500-800 $\mu$atm | 5 | 181557.2 | <0.0001 | No effect |
| 801-1000 $\mu$atm | 3 | 19.1 | 0.0003 | No effect |
| 1001-1500 $\mu$atm | 4 | 137.2 | <0.0001 | Negative |
| 1501-2000 $\mu$atm | 4 | 11910.7 | <0.0001 | Negative |
| >2000 $\mu$atm | 1 | 9.2 | 0.0024 | Negative |
| | | | | |
| Behaviour | | | | |
| 500-800 $\mu$atm | 0 studies | N/A | | |
| 801-1000 $\mu$atm | 1 | 35.4 | <0.0001 | No effect |
| 1001-1500 $\mu$atm | 0 studies | N/A | | |
| 1501-2000 $\mu$atm | 0 studies | N/A | | |
| >2000 $\mu$atm | 0 studies | N/A | | |
| | | | | |
| Feeding | | | | |
| 500-800 $\mu$atm | 0 studies | N/A | | |
| 801-1000 $\mu$atm | 1 | 21.4 | <0.0001 | No effect |
| 1001-1500 $\mu$atm | 3 | 45.3 | <0.0001 | No effect |
| 1501-2000 $\mu$atm | 0 studies | N/A | | |
| >2000 $\mu$atm | 0 studies | N/A | | |

Table S.5 continued.

| | Q-Test Results | | | Model Results |
|---|---|---|---|---|
| | df | Q | p | |
| *per $CO_2$ Level and Biological Measurement continued* | | | | |
| Growth Rate | | | | |
| 500-800 $\mu$atm | 1 study | N/A | | No effect |
| 801-1000 $\mu$atm | 2 | 0.2 | **0.8853** | No effect |
| 1001-1500 $\mu$atm | 2 | 0.2 | **0.8945** | No effect |
| 1501-2000 $\mu$atm | 0 studies | N/A | | |
| >2000 $\mu$atm | 0 studies | N/A | | |
| | | | | |
| Lipid and Protein Content | | | | |
| 500-800 $\mu$atm | 0 studies | N/A | | |
| 801-1000 $\mu$atm | 1 | 0.4 | **0.5191** | Negative |
| 1001-1500 $\mu$atm | 0 studies | N/A | | |
| 1501-2000 $\mu$atm | 0 studies | N/A | | |
| >2000 $\mu$atm | 0 studies | N/A | | |
| | | | | |
| Shell State | | | | |
| 500-800 $\mu$atm | 1 | 1.1 | **0.2923** | Negative |
| 801-1000 $\mu$atm | 2 | 14.5 | 0.0007 | No effect |
| 1001-1500 $\mu$atm | 1 study | | N/A | Negative |
| 1501-2000 $\mu$atm | 0 studies | N/A | | |
| >2000 $\mu$atm | 4 | 11.035 | 0.0262 | Negative |
| | | | | |
| Survival | | | | |
| 500-800 $\mu$atm | 0 studies | N/A | | |
| 801-1000 $\mu$atm | 1 study | N/A | | Negative |
| 1001-1500 $\mu$atm | 2 | 63.5 | <0.0001 | Negative |
| 1501-2000 $\mu$atm | 0 studies | N/A | | |
| >2000 $\mu$atm | 0 studies | N/A | | |

Table S6: Q test and random effects model results for fish.

| | Q-Test Results | | | Model Results |
|---|---|---|---|---|
| | df | Q | p | |
| *per $CO_2$ Level* | | | | |
| All levels | 23 | 1428.2 | <0.0001 | No effect |
| 500-800 $\mu$atm | 4 | 6.4 | **0.1701** | Positive |
| 801-1000 $\mu$atm | 10 | 116.6 | <0.0001 | No effect |
| 1001-1500 $\mu$atm | 4 | 110.2 | <0.0001 | No effect |
| >2000 $\mu$atm | 2 | 31.6 | <0.0001 | Negative |
| | | | | |
| *per Biological Measurement* | | | | |
| Development | 1 | 1102.2 | <0.0001 | Positive |
| CS | 7 | 73.9 | <0.0001 | No effect |
| Growth Rate | 1 study | N/A | | Negative |
| Heart Rate | 1 | 6.4 | 0.0115 | No effect |
| Oxygen Consumption | 8 | 221.5 | <0.0001 | No effect |
| | | | | |
| *per $CO_2$ Level and Biological Measurement* | | | | |
| Development | | | | |
| 500-800 $\mu$atm | 1 study | N/A | | Positive |
| 801-1000 $\mu$atm | 1 study | N/A | | Positive |
| 1001-1500 $\mu$atm | 0 studies | N/A | | |
| >2000 $\mu$atm | 0 studies | N/A | | |
| | | | | |
| Citrate synthase enzyme activity (CS) | | | | |
| 500-800 $\mu$atm | 1 study | N/A | | No effect |
| 801-1000 $\mu$atm | 4 | 65.3 | <0.0001 | No effect |
| 1001-1500 $\mu$atm | 1 study | N/A | | No effect |
| >2000 $\mu$atm | 0 studies | N/A | | |
| | | | | |
| Growth Rate | | | | |
| 500-800 $\mu$atm | 0 studies | N/A | | |
| 801-1000 $\mu$atm | 1 study | N/A | | Negative |
| 1001-1500 $\mu$atm | 0 studies | N/A | | |
| >2000 $\mu$atm | 0 studies | N/A | | |
| | | | | |
| Heart Rate | | | | |
| 500-800 $\mu$atm | 1 study | N/A | | Positive |
| 801-1000 $\mu$atm | 1 study | N/A | | No effect |
| 1001-1500 $\mu$atm | 0 studies | N/A | | |
| >2000 $\mu$atm | 0 studies | N/A | | |
| | | | | |
| Oxygen Consumption | | | | |
| 500-800 $\mu$atm | 0 studies | N/A | | |
| 801-1000 $\mu$atm | 2 | 4.0 | **0.1341** | Positive |
| 1001-1500 $\mu$atm | 3 | 106.0 | <0.0001 | No effect |
| >2000 $\mu$atm | 1 | 11.0 | 0.0009 | Negative |
